# Supplementary figures and images for: Multiple systems in macaques for tracking prediction errors and other types of surprise
Source: PLoS Biol. 2020 Oct 30;18(10):e3000899. doi: 10.1371/journal.pbio.3000899 (PMC7657565; doi:10.1371/journal.pbio.3000899)

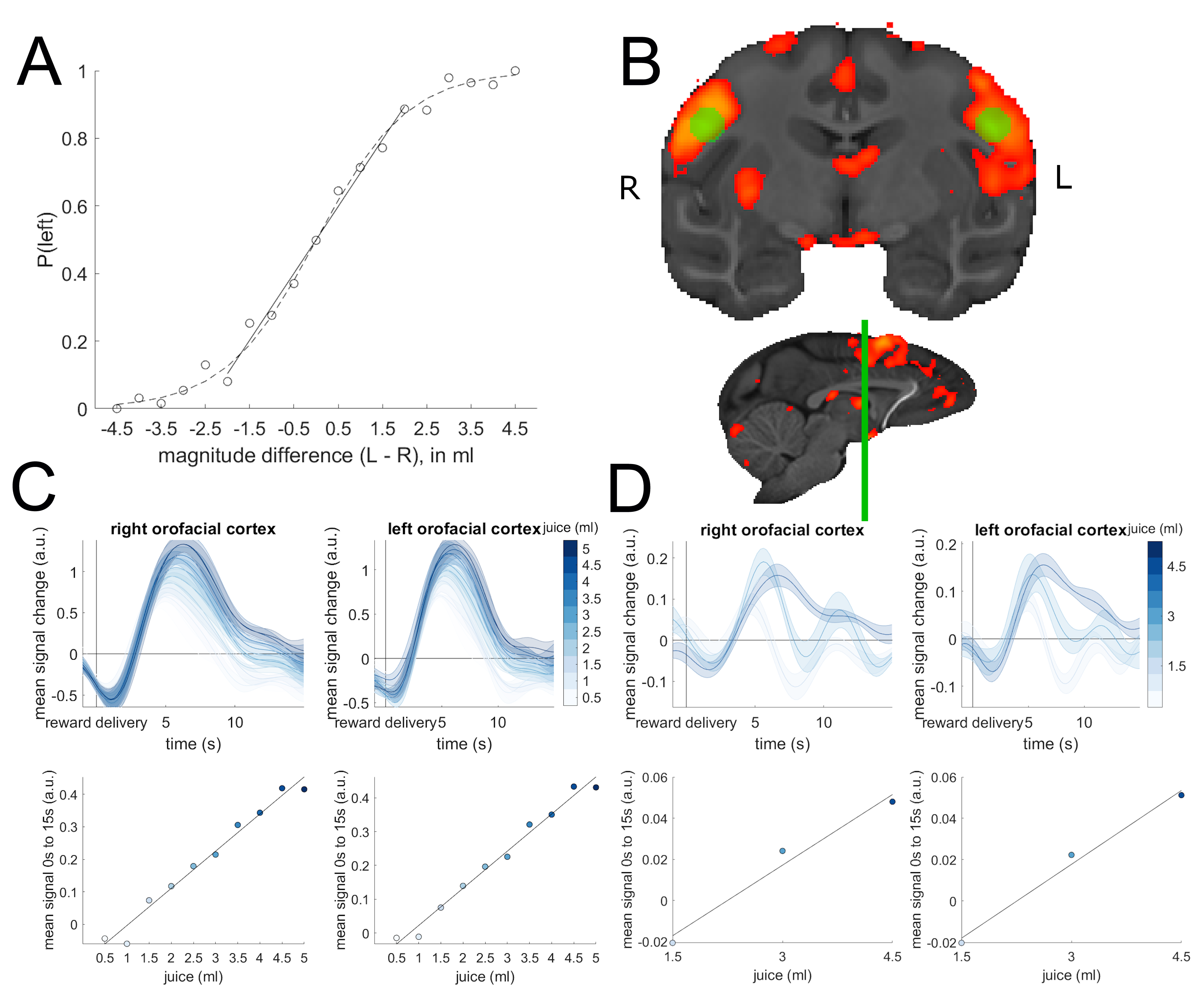

Supplement: S1 Fig — To validate that monkeys can reliably distinguish between juice amounts of 1.5 ml, we re-analyzed data from a visual discrimination task. In this task, monkeys (n = 4) had to select one of 2 displayed stimuli that were probabilistically rewarded with a juice amount between 1 and 10 drops (0.5 ml per drop). The color of the stimuli varied in shade between blue and green. (A) The proportion of times the left stimulus was chosen as a function of the difference in reward magnitude (in ml) between the left and the right stimulus. For the slope of the curve in the central region (between magnitude differences of −2 ml and 2 ml), we can see that discrimination performance improves by 9.89% on average per drop (solid line; t(7) = 23.08, p < 0.001). (B) The thresholded and cluster-corrected map of activity covarying with reward from a whole-brain analysis of this task (shown with a threshold of z = 4.5). The cluster shows prominent bilateral activity in the orofacial sensorimotor cortex. We placed spherical ROIs at the most posterior local maxima in both the left and right orofacial activity (i.e., in the somatosensory cortex indicated by green ROIs; F99 coordinates 22.6, −4.02, 11.1 and −23.1, −3.51, 11.1) and extracted the BOLD time courses. (C) The extracted time courses from the ROIs indicated in (B) split by the amount of juice received (from 0.5 ml to 5 ml in 0.5-ml intervals) after reward delivery (top) and averaged over a window of 15 s (bottom). BOLD activity becomes stronger the more juice monkeys received in both the right X2(1) = 20.38, p<0.001) and left X2(1) = 17.687, p<0.001) orofacial somatosensory cortex. (D) The extracted time course from the same (now a priori) ROIs for the present study, again split up by the juice amount the monkeys received (1.5 ml, 3 ml, and 4.5 ml). As can be seen, BOLD signals are larger the more juice the monkey receives in both the right X2(1) = 8.892, p = 0.003) and left X2(1) = 10.984, p<0.001) orofacial somatosensory cortex. We [file pbio.3000899.s001.png]

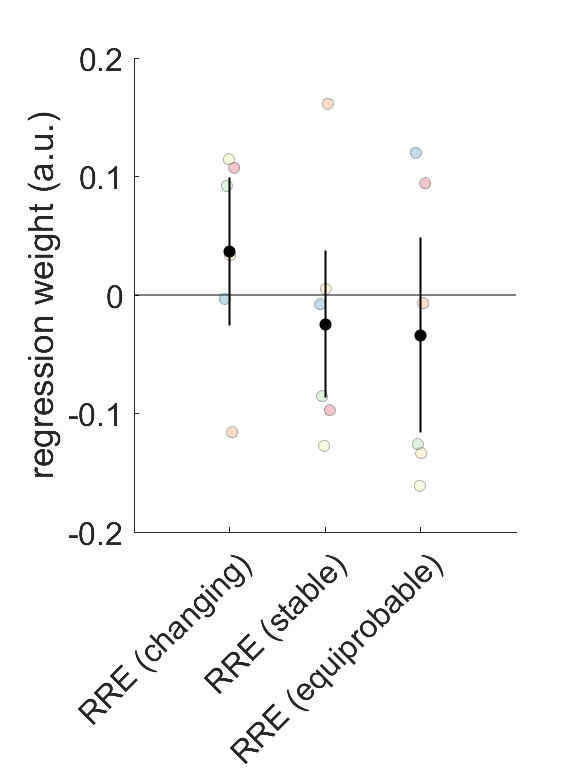

Supplement: S2 Fig — To assess whether behavioral RRE effects were modulated by session type, we ran GLME1 separately for changing/learnable, stable/unlearnable, and equiprobable sessions. The effect was not significant in changing/learnable sessions (X2(1) = 0.318, p = 0.573; left column), stable/unlearnable sessions (X2(1) = 0.150, p = 0.699; middle column), or equiprobable sessions (X2(1) = 0.134, p = 0.714; right column). Data and code to reproduce the figure can be found at https://doi.org/10.5281/zenodo.3993116. (PNG) [file pbio.3000899.s002.png]

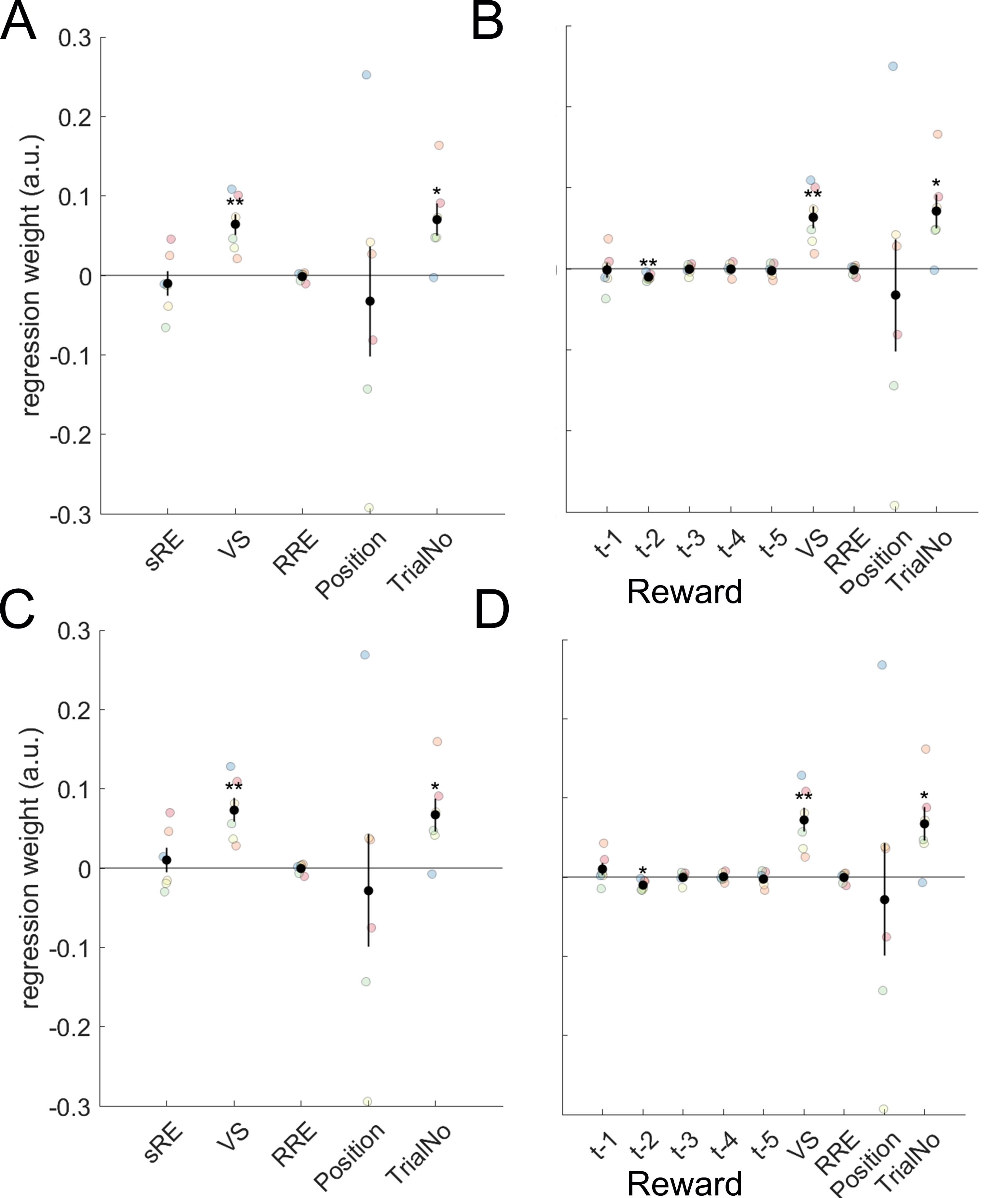

Supplement: S3 Fig — We also ran GLMEs with RTs as the dependent variable that contained the same independent variables as GLME1 and GLME2. For GLME3 (A) and GLME4 (B), outlier trials were excluded. GLME3 and GLME4 only differed in that in GLME3, the rewards from the previous 5 trials are combined into the sRE using a learning rate estimated from GLME2 as described in the main text. In GLME3, VS was significant (X2(1) = 9.409, p = 0.002), whereas sRE (X2(1) = 0.433, p = 0.511) and RRE (X2(1) = 0.185, p = 0.667) were not. In GLME5 (C) and GLME6 (D), we excluded all outlier, error, and repeat trials (trial after an error). Again, in GLME5, VS was significant (X2(1) = 9.631, p = 0.002), whereas sRE (X2(1) = 0.444, p = 0.505) and RRE (X2(1) = 0.030, p = 0.862) were not. Data and code to reproduce the figure can be found at https://doi.org/10.5281/zenodo.3993116. (PNG) [file pbio.3000899.s003.png]

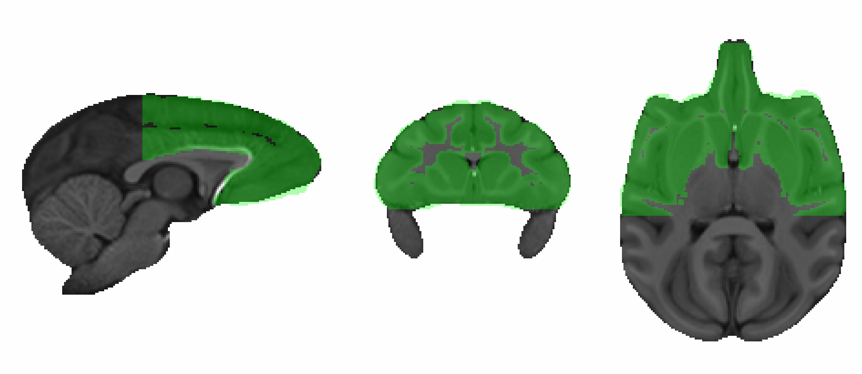

Supplement: S4 Fig — As we were primarily interested in prefrontal cortex and striatum and the nature of their PEs, we specified a VOI covering both. All whole-brain analyses in the main text were carried out using this VOI. (PNG) [file pbio.3000899.s004.png]

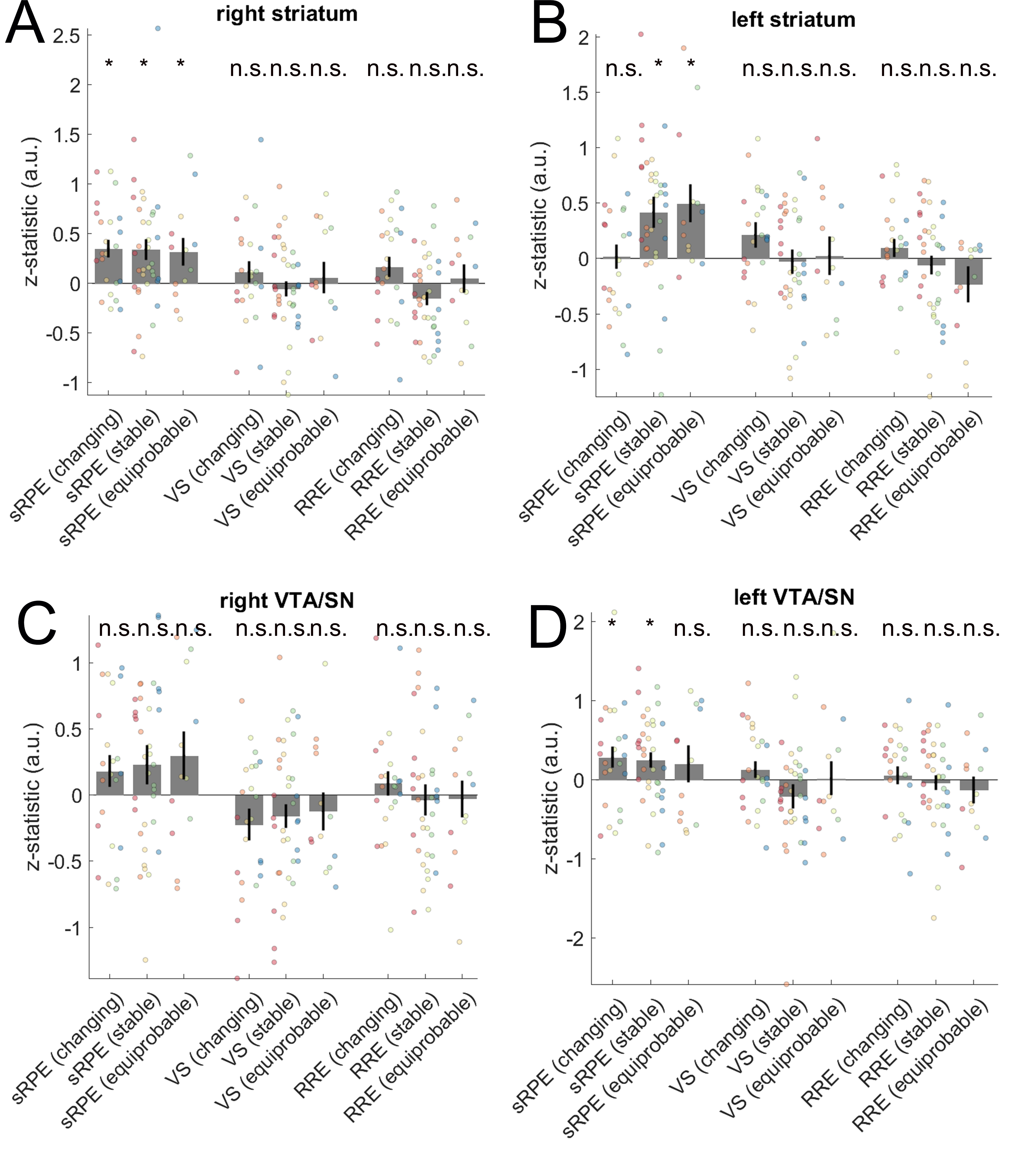

Supplement: S5 Fig — We examined the extracted z-statistics shown in Fig 3, here split up by session type. Labelling conventions are the same as in Fig 3. Data and code to reproduce the figure can be found at https://doi.org/10.5281/zenodo.3993116. (PNG) [file pbio.3000899.s005.png]

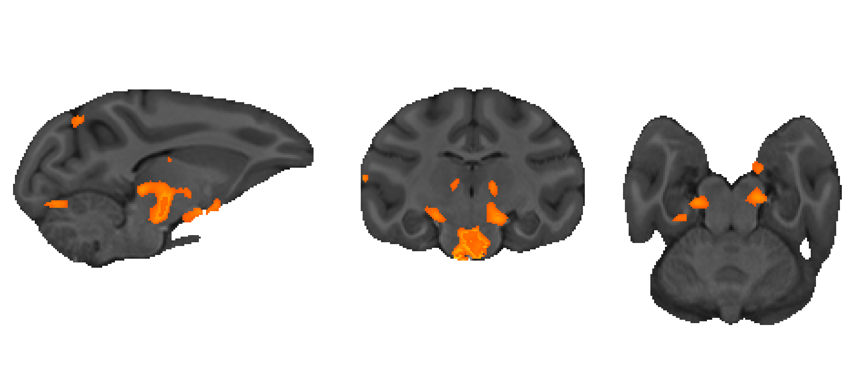

Supplement: S6 Fig — When running a cluster correction on the whole brain (without the VOI) for our sRPE regressor we also found activity in the dopaminergic midbrain. The precise location of these clusters can be found in S6 Table. Data to reproduce the figure can be found at https://doi.org/10.5281/zenodo.3993116. (PNG) [file pbio.3000899.s006.png]

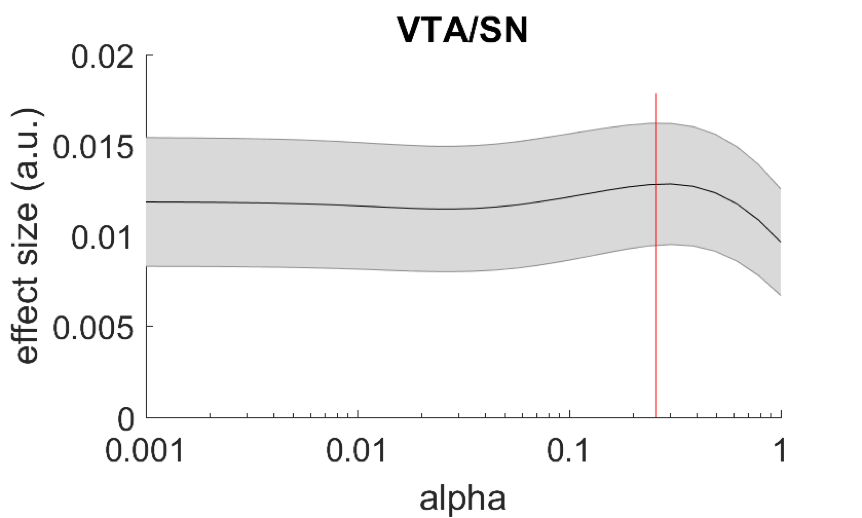

Supplement: S7 Fig — To consider the possibility that sRPE signals might be an artefact of the learning rate we used when estimating the monkeys’ reward value expectations, we examined the BOLD response in the VTA/SN ROI further. By running a linear regression on the extracted BOLD time course time-locked to reward delivery (with terms coding for a constant, sRPE, VS, RRE, and trial number), we can determine the effect size of the sRPE regressor when constructing it using different learning rates. Effect size is here defined as the averaged beta weights for regressions run on the 10 s after reward delivery. The red line indicates the learning rate we used in the main text (0.257). As can be seen, the empirical learning rate is close to the peak effect strength in VTA/SN, but the effect strength is positive throughout regardless of learning rate. Data and code to reproduce the figure can be found at https://doi.org/10.5281/zenodo.3993116. (PNG) [file pbio.3000899.s007.png]

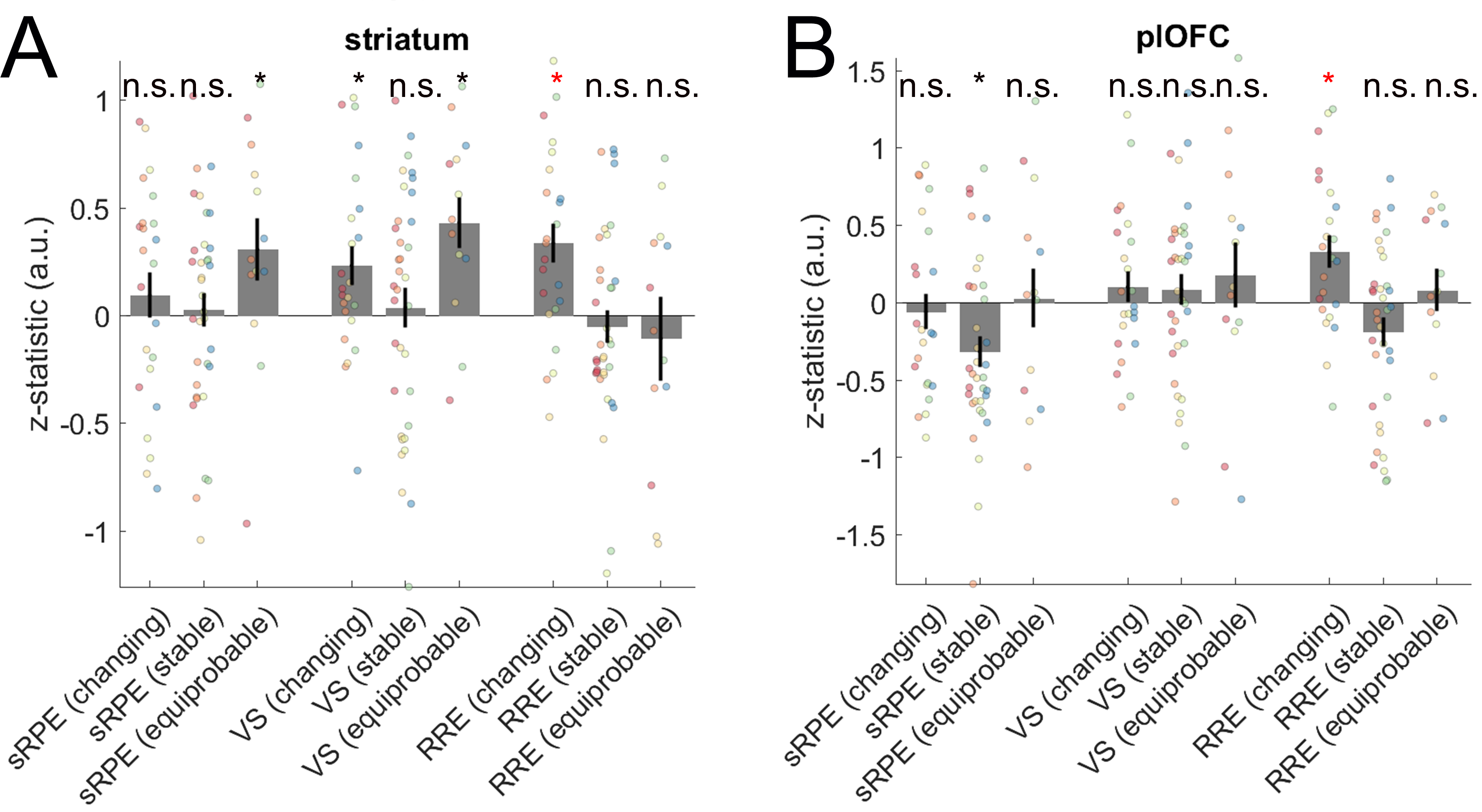

Supplement: S8 Fig — The z-statistics shown in Fig 4 split up by session type, including the equiprobable sessions in anterior lateral striatum and plOFC. Labelling conventions are the same as in Fig 3. Data and code to reproduce the figure can be found at https://doi.org/10.5281/zenodo.3993116. (PNG) [file pbio.3000899.s008.png]

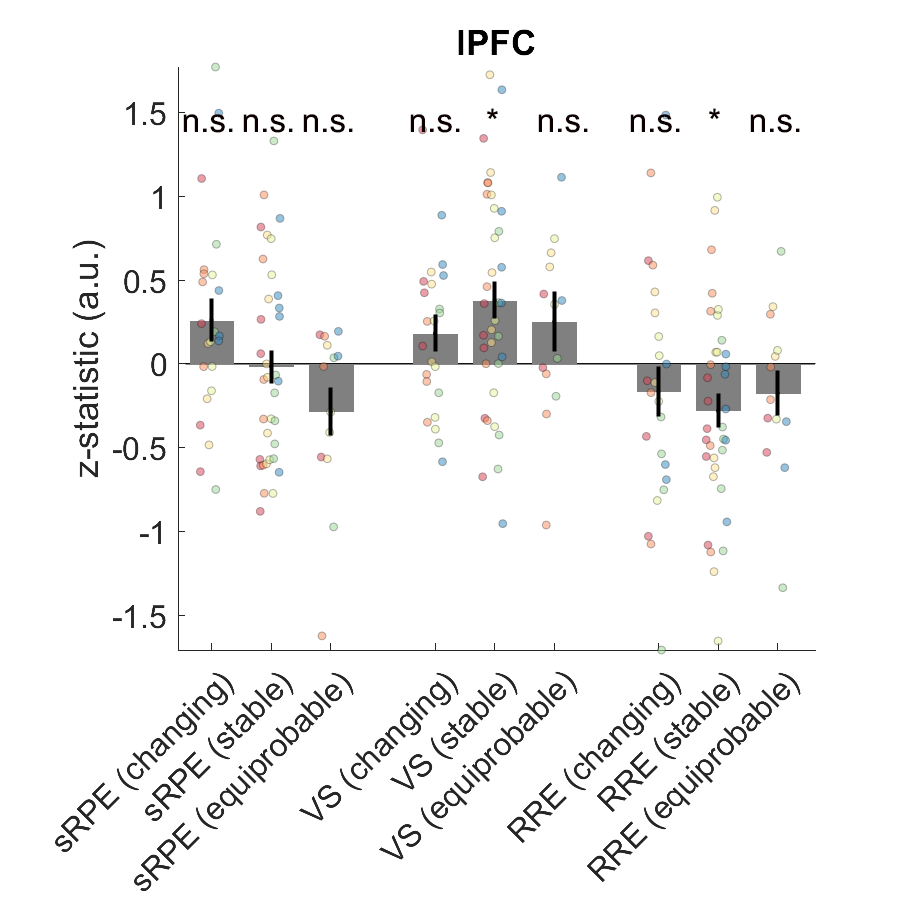

Supplement: S9 Fig — We also examined the extracted z-statistics shown in Fig 5 split up by session type. Labelling conventions are the same as in Fig 3. Data and code to reproduce the figure can be found at https://doi.org/10.5281/zenodo.3993116. (PNG) [file pbio.3000899.s009.png]

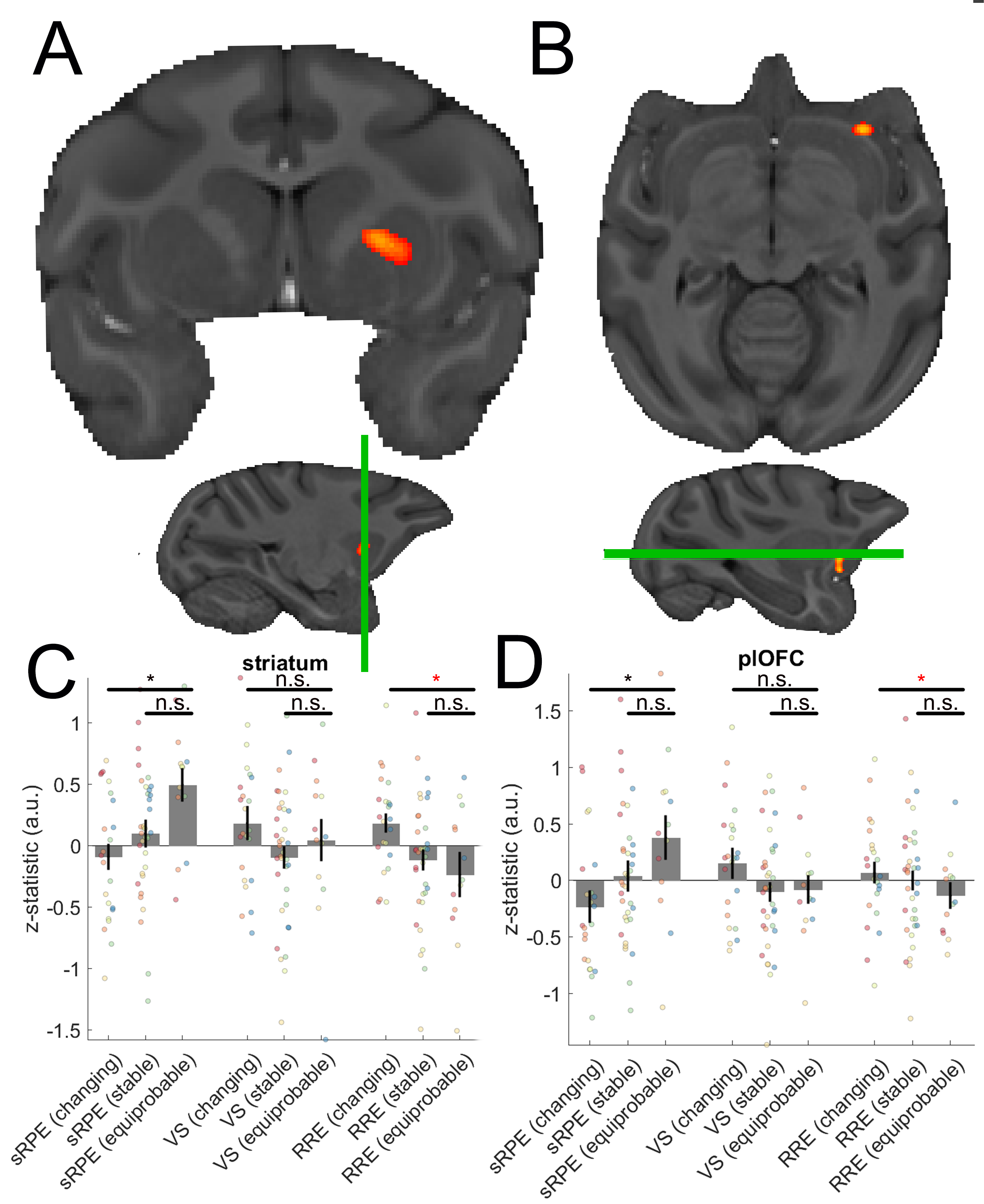

Supplement: S10 Fig — As an alternative to the analysis shown in Fig 4, we also examined RRE by comparing changing/learnable and stable/unlearnable sessions against the equiprobable control sessions. (A) On the whole-brain level, we found significant activity in the striatum for a contrast comparing changing/learnable and equiprobable sessions. (B) We also found activity in the plOFC for the contrast comparing changing/learnable and equiprobable sessions although now the activity was even more centered on the boundary with the anterior insula. All local maxima of the cluster are shown in S4 Table. (C) Extracting the z-statistics from an ROI placed at the peak activity illustrates the difference for RRE between changing/learnable and stable/unlearnable sessions in the striatum (third column from the right and second column from the right). We did not find a significant difference between stable/unlearnable and equiprobable sessions for RRE (X2(1) = 0.516, p = 0.474). Our analysis revealed a significant difference between changing/learnable and equiprobable sessions for sRPE in the striatum (X2(1) = 7.071, p = 0.008) but with a different sign. For sRPE, the difference between stable/unlearnable and equiprobable was not significant (X2(1) = 3.160, p = 0.076). For VS, neither the difference between changing/learnable and equiprobable (X2(1) = 0.110, p = 0.415) nor between stable/unlearnable and equiprobable sessions (X2(1) = 0.516, p = 0.472) was significant. (D) In the lOFC, we again did not find a significant difference between stable/unlearnable and equiprobable sessions for RRE (X2(1) = 0.665, p = 0.415). For sRPE, there again was a significant difference between changing/learnable and equiprobable sessions in the opposite direction (X2(1) = 4.007, p = 0.045). The difference between changing/learnable and equiprobable sessions was not significant for sRPE (X2(1) = 2.289, p = 0.130). For VS, again neither the difference between changing/learnable and equiprobable (X2(1) = 2.552, p = 0.110 [file pbio.3000899.s010.png]

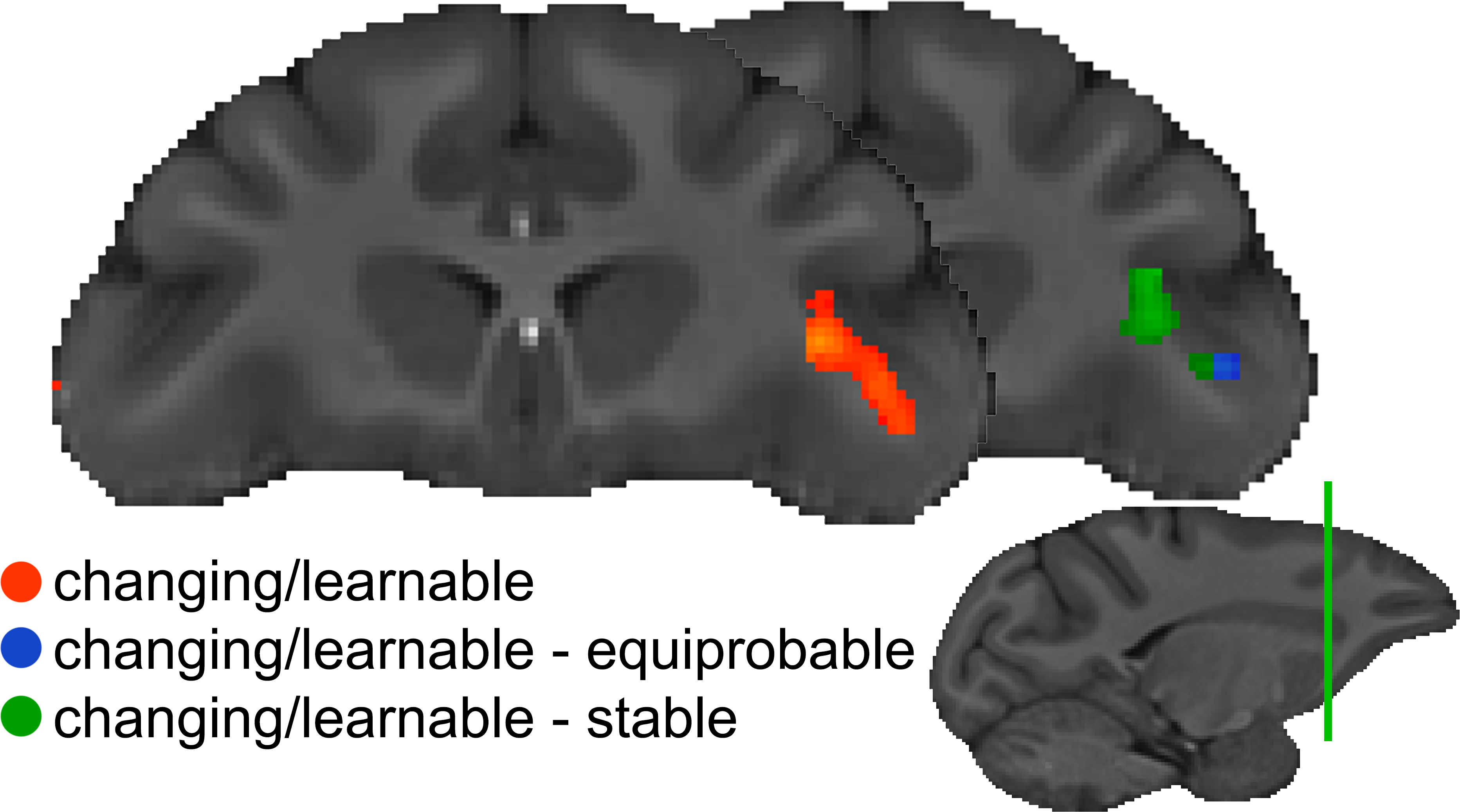

Supplement: S11 Fig — RRE activity in plOFC in changing/learnable sessions (red) is located in the same area as activity when we contrast changing/learnable and equiprobable sessions (blue) or changing/learnable and stable sessions (green). All effects shown here are at a threshold of 2 and are shown without applying any cluster correction. Data and code to reproduce the figure can be found at https://doi.org/10.5281/zenodo.3993116. (PNG) [file pbio.3000899.s011.png]

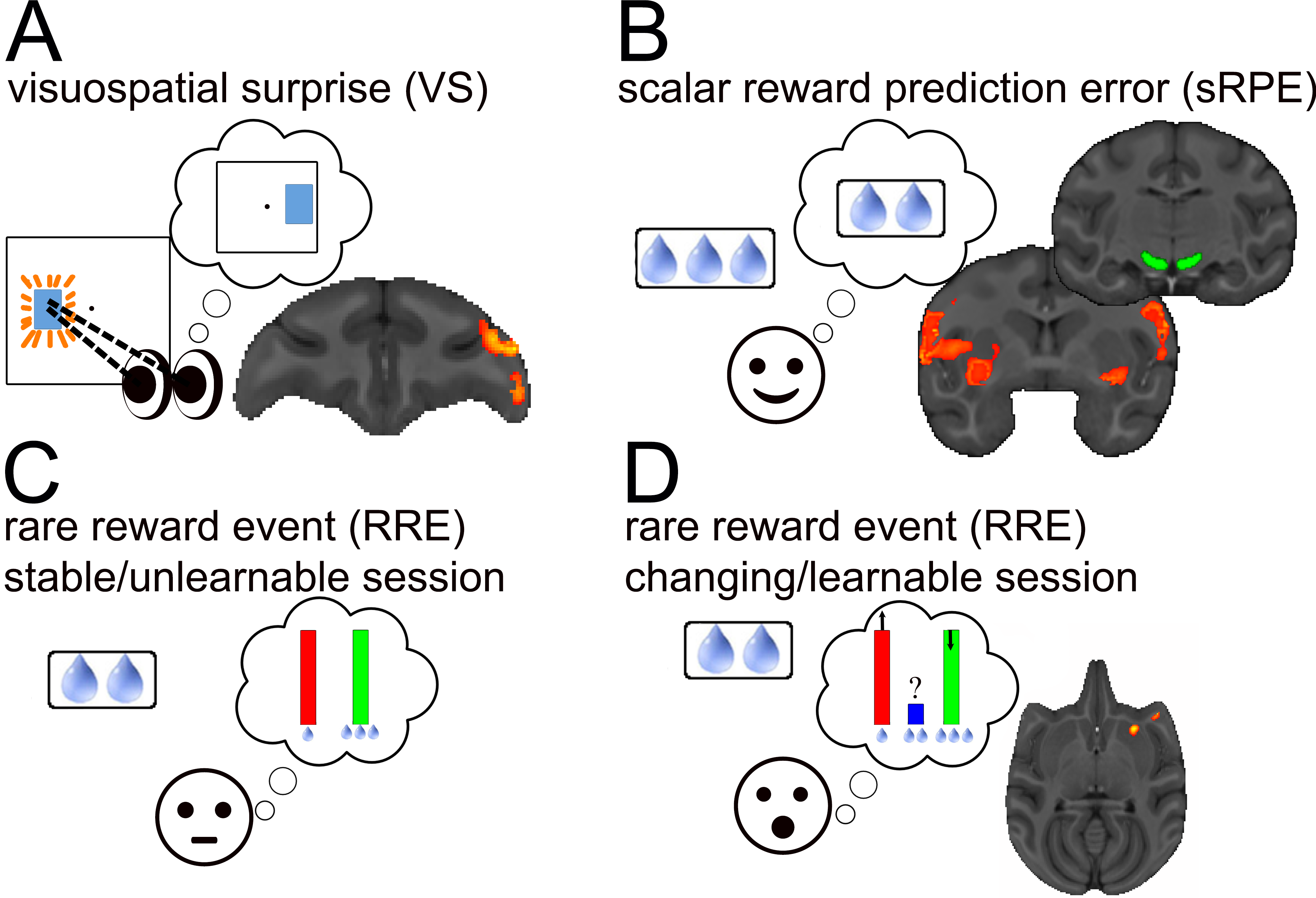

Supplement: S12 Fig — (A) VS occurred when the stimulus is expected to occur on one side of the screen (thought bubble) but surprisingly appears on the other side of the screen. We found activity in the posterior lPFC in response to VS. (B) sRPEs occurred when the macaque experienced a reward level that was higher or lower than its reward expectation (thought bubble). We found activity in the dopaminergic midbrain and ventrolateral striatum in response to sRPE. (C) RRE occurred when an infrequent reward (2 drops) was sampled. We found no neural activity for RRE results in in stable/unlearnable sessions. This result might be because in such sessions less monitoring of reward occurrences takes place because occurrences of 1 or 3 drops cannot be predicted (thought bubble). (D) In contrast, we found activity for RRE in lateral striatum and plOFC in changing/learnable sessions. This might be because in such sessions the frequency of 1 and 2 drops of juice is actively tracked to estimate the current average reward rate, rendering obtaining 2 drops of juice a potential event of interest that is encoded as a new task relevant state (thought bubble). (PNG) [file pbio.3000899.s012.png]
