# Supplementary material for: Multiple systems in macaques for tracking prediction errors and other types of surprise
Source: PLoS Biol. 2020 Oct 30;18(10):e3000899. doi: 10.1371/journal.pbio.3000899 (PMC7657565; doi:10.1371/journal.pbio.3000899)
Supplement: Attachment — Submitted filename: reply_to_reviewers.docx [file pbio.3000899.s020.docx]

We are grateful for the comments from all three reviewers. Their insightful suggestions helped us improve our manuscript considerably and we are happy to present our updated results here. Below we are responding to each comment in turn. The reviewers’ original points are shown in italics while our responses are shown in blue ink. Sections of the manuscript and supplementary information are shown in a standard font with new areas highlighted in red.

*Reviewer #1: This manuscript describes a non-human primate fMRI study focusing on neural responses to different types of prediction errors. The elegant experimental design dissociates signed reward prediction errors (sRPE), visuospatial surprise (VS) and responses to rare events (RRE). In line with previous human fMRI and animal recording studies, sRPE correlate with activity in the ventral striatum and midbrain. However, these areas did not respond to VS and RRE. In contrast, VS trials evoked activity changes in lateral PFC, whereas RRE evoked activity in the lateral striatum and posterior lateral OFC, but only in sessions in which the probability of the two frequent events changed across trials. The results described here are both important and interesting. The study is well-designed and executed, and the manuscript is very well-written. However, there are two potential issues with the experimental design that should be addressed. In addition, I do not think the discussion is currently providing a balanced review of the literature and the findings.*

Thank you for your feedback and kind words. We hope you feel that our answers to the issues you raised are appropriate.

*Major points*

*1. The authors present behavioral evidence that subjects can distinguish between 1 and 3 drops of juice. However, there is no evidence that they can detect the difference between 1 and 2, and 2 and 3 drops, especially in the stable sessions. This has implications for the interpretations of the imaging findings. First, if subjects cannot detect this difference, there may be no brain response. This could explain the null results in the RRE condition, and thus, a failure of the experimental manipulation to induce the intended effect needs to be taken into consideration in order to avoid prematurely rejecting the H1 (i.e., the results could have come out very differently if the experiment had used 1, 3, and 5 drops). In addition, 2 drops could have been perceived as more ambiguous and thus responses to 2 drops may differ in other important aspects (e.g., lower confidence) which needs to be considered when interpreting these results.*

We strongly agree with the reviewer that this is an important point. In fact, this comment relates to a broader issue in researching reward-guided decision-making in macaques, namely which reward sizes monkeys can reliably distinguish. In the present study 1 drop of juice corresponds to 1.5ml but in previous work from our own group we used even smaller reward sizes. For example, in (Khalighinejad et al., 2020, Neuron) monkeys also received either 1, 2 or 3 drops of juice but each drop of juice consisted of 1ml. Other labs have used even smaller drop sizes: for example, (Tobler et al., 2005, Science) used drops of 0.05ml, 0.15ml, and 0.50ml.

The reason we believe monkeys can reliably distinguish between such juice amounts is that we previously, in an unpublished study, established that in a visual discrimination task monkeys can distinguish between drops as small as 0.5ml. In the task, two stimuli were shown on screen that varied in shade between blue and green. Each stimulus was associated with a probabilistic juice amount between 1-10 drops (0.5ml per drop), and monkeys (n=4) were trained to choose between the two stimuli. We ran a whole brain analysis which included one regressor coding for the amount of received reward. The results are shown below.

As can be seen, peaks of activity are prominent in the left and right orofacial sensorimotor cortex. We examined activity in regions of interest (ROIs) centred on the most posterior sub-peak in both the left and the right hemisphere as this is the most likely location of the somatosensory part of orofacial-associated cortex. When we extract the BOLD timecourse from these orofacial activity peaks for each of the 10 reward amounts, we see that the average activity in this area scales linearly with the number of drops received.

Using these independently obtained coordinates for orofacial sensorimotor areas that code for reward amount in the previous study, we can also extract the BOLD timecourse for 1, 2, and 3 drops of juice in the task presented in our manuscript. This is what we show below.

As can be seen once again, activity in the orofacial sensorimotor cortex is stronger for a larger juice amount. The activity shown here, suggests that at this “low” sensorimotor level there are reliable and replicable differences in how the brain responds to rewards that varies in amount. In fact, even smaller differences in reward sizes than the ones that we employ in the current study are associated with different neural responses. This demonstrates that information about reward amounts of the size we employ is thus readily available to other parts of the brain that encode other reward and prediction error features. This is the case in the current experiment just as it was in the previous experiments.

Notably the previous experiment, because it employed a very different task design allowing for binary choices, also showed, at a behavioural level, that macaques were able to learn to discriminate between colours associated with these different levels of reward. Below, we show a psychometric curve that illustrates the discrimination performance (choices of the left or right hand stimulus) as a function of the number of juice drops (0.5mL in size – in other words, one third of the size of the drops in the current experiment). From visual inspection it is clear that addition or removal of a single drop impacts on discrimination. For example, if we consider the slope of the curve in the central region (between a magnitude difference of -2ml and 2ml), we can see that discrimination performance improves by 9.89% on average per drop and that macaques are more likely to be right than wrong when choosing between options associated with reward levels that differ by just one drop size. (t(7)=23.08, p<0.001).

In the revised manuscript we have presented the recordings of activity from the orofacial sensorimotor cortex from current task in the main manuscript in figure 3. We have explained that the recordings are taken from an ROI that was derived from a previous study in which it was possible to observe linearly changing activity levels as drops a third of the size were increased in number and we have shown these re-analyzed data in a new figure in the supplementary figure S1. In the same figure we also show that animals learned to discriminate between colours associated with these different reward drop numbers. The revised text now includes the following sentence in the Results section:

“Animals received a juice reward of one, two, or three drops after touching the response sensor on the corresponding side of the stimulus (Fig 1A). To first validate that monkeys can reliably distinguish between such juice amounts, we re-analyzed data from a different task and showed that that was indeed the case when both behavioral and neural data were analyzed (Fig S1)”.

The figure legend for supplementary figure S1 now reads:

Fig. S1. To validate that monkeys can reliably distinguish between juice amounts of 1.5ml, we re-analysed data from a visual discrimination task. In this task monkeys (n=4) had to select one of two displayed stimuli that were probabilistically rewarded with a juice amount between 1-10 drops (0.5ml per drop). The colour of the stimuli varied in shade between blue and green. **(A)** The proportion of times the left stimulus was chosen as a function of the difference in reward magnitude (in ml) between the left and the right stimulus. For the slope of the curve in the central region (between magnitude differences of -2 ml and 2 ml) we can see that discrimination performance improves by 9.89% on average per drop (solid line; t(7)=23.08, p<0.001). **(B)** The thresholded and cluster-corrected map of activity covarying with reward from a whole-brain analysis of this task (shown with a threshold of z=4.5). The cluster shows prominent bilateral activity in the orofacial sensorimotor cortex. We placed spherical ROIs at the most posterior local maxima in both the left and right orofacial activity (i.e. in the somaotosensory cortex indicated by green ROIs; F99 coordinates 22.6, -4.02, 11.1 and -23.1, -3.51, 11.1) and extracted the BOLD timecourses. **(C)** The extracted timecourses from the ROIs indicated in (B) split by the amount of juice received (from 0.5ml to 5ml in 0.5ml intervals) after reward delivery (top) and averaged over a window of 15s (bottom). BOLD activity becomes stronger the more juice monkeys received in both the right $X^{2}\left( 1 \right)=20.38, p<0.001$) and left $X^{2}\left( 1 \right)=17.687, p<0.001$) orofacial somatosensory cortex. **(D)** The extracted timecourse from the same (now *a priori*) ROIs for the present study, again split up by the juice amount the monkeys received (1.5ml, 3ml and 4.5ml). As can be seen, BOLD signals are larger the more juice the monkey receives in both the right $X^{2}\left( 1 \right)=8.892, p=0.003)$ and left $X^{2}\left( 1 \right)=10.984, p<0.001)$ orofacial somatosensory cortex. We thus conclude that our monkeys can reliably distinguish between 1, 2 and 3 drops of juice and that distinct activity patterns associated with each outcome time are available as inputs to any neural learning mechanism in the present study. The averaged BOLD timecourse for both the left and right orofacial area is also shown in Figure 3E in the main text.

*2. The design confounds sRPE with reward size. This is evident in the somatosensory responses observed in Fig. 3A. As the authors are well aware, one way to dissociate RPE from reward size is to compare responses to the same reward size between trials with different expectations. Because expectations in the current experiment were relatively constant across all trials (given the small learning rate), this may be difficult to achieve here. Nevertheless, the authors could estimate the effects of a parametric sRPE regressor that is orthogonalized with respect to reward size and test whether the results are similar. In addition, expectations change more strongly in the learnable sessions, so those sessions may provide another avenue to address this issue.*

As the reviewer rightly points out here, we describe an effect of sRPE in the manuscript but never disassociate it from reward amount, with which it is highly confounded. The reviewer suggests a number of ways in which we could try to disassociate these two effects but acknowledges themselves that these analyses might be difficult for our task. Thus, instead of attempting the suggestions of the reviewer, we would like to offer an alternative analysis that we hope is both simpler and more direct and suggests that our effects are indeed due to sRPEs.

Our analysis is based on the idea that throughout all of our session types two drops of juice was designed to be the average juice amount, while one drop was worse (thus evoking a negative sRPE) and three drops was better (thus evoking a positive sRPE) than the baseline. Therefore, we can simply plot the average BOLD timecourse after receiving 1, 2, or 3 drops of juice in our ROIs (we collapse over the left and right hemisphere here).

As can be seen from the above figure, an area that purely processes reward amount, such as the orofacial sensorimotor cortex, shows an initial positive response for 1, 2, and 3 drops of juice. In contrast, areas that are commonly associated with processing sRPEs show suppressed activity after receiving 1 drop of juice and positive activity after receiving 3 drops of juice. We hope that our results suggest that the striatum or VTA/SN are regions that do not simply care about the amount of reward received, as this would mean that they should also have a positive response after receiving 1 drop of juice and response profile should resemble that of the orofacial cortex. Moreover, we would also like to point out that there is a large body of literature that supports the idea of sRPE coding in VTA/SN and striatum. Thus, this simple analysis, therefore, illustrates an effect that is consistent with many other studies in which activity in the dopaminergic midbrain and striatum was found to encode not simply reward amount but a sRPE, rather than being the only evidence. It is also important to point out that our design was not further optimized to dissociate between scalar reward and reward prediction signal precisely because it is so uncontroversial and we wanted to have a relatively stable expectation to test our primary hypothesis contrasting such scalar prediction error signals with other types of error signals. For such a comparison we can remain agnostic about whether areas such as VTA/SN and striatum encode scalar reward or scalar RPE, because we can show that the parts of the striatum that carry either/both do not carry the other signals, while other brain regions such as posterior OFC doesn’t carry either scalar reward or prediction signals but it does carry a rare reward event signal. In the revised manuscript we have made the following changes to clarify the three different patterns of response in the three different areas:

- The above three BOLD timecourses have been added to figure 3 and the figure legend for them reads:

**(E)** BOLD timecourses extracted from three ROIs in the ventrolateral striatum, the SN/VTA and the orofacial cortex. The timecourses are averaged over both hemispheres. For the location of the orofacial cortex ROI see Fig S1. Separate timecourses are shown for each of the three levels of reward that the monkey received (1, 2 or 3 drops). The orofacial area processes reward amount and thus all three timecourses initially move in a positive direction before peaking. In contrast, ventrolateral striatum and SN/VTA process sRPEs and thus, in both cases, the timecourse for receiving one drop of juice, which is associated with a negative sRPE, results in supressed activity.

- In the results section we have added the following paragraph outlining these results:

To illustrate these results, we extracted BOLD timecourses from the ROIs in the ventrolateral striatum, the SN/VTA and a control region in the orofacial cortex (Fig 3E; see Fig S1 for the location of the control region). When separating the timecourses of activity as a function of the amount of juice received (1, 2, or 3 drops) we observed suppressed activity after receiving one drop in the ventrolateral striatum ($X^{2}\left( 1 \right)=10.219, p=0.001$) and the SN/VTA ($X^{2}\left( 1 \right)=6.250, p=0.012$), which is due to the negative sRPE the monkeys experience. After receiving three drops of juice, which is associated with a positive sRPE, we observe enhanced activity in the ventrolateral striatum ($X^{2}\left( 1 \right)=8.954, p=0.003$) and the SN/VTA ($X^{2}\left( 1 \right)=10.191, p=0.001$). In contrast, an area that processes reward amount such as the orofacial somatosensory cortex shows a different activity profile: We observe no significant negative change in activity in orofacial somatosensory cortex. In fact, if anything, the response is positive (although this positive change does not reach a level of statistical significance because the initial positive is later suppressed ($X^{2}\left( 1 \right)=2.1.03, p=0.147$) but we observe positive activity after receiving two ($X^{2}\left( 1 \right)=8.931, p=0.003$) or three ($X^{2}\left( 1 \right)=13.463, <0.001$) drops of juice.

- In the Material and Methods section we added a paragraph detailing our procedure for analyzing BOLD timecourses:

When effects were illustrated with extracted BOLD timecourses, these timecourses were extracted from the same ROIs and were up-sampled by a factor of 10 using spline interpolation. To test for statistical significance, we average the timecourses of each session over time (from 0s to 10s in Fig 3 and from 0s to 15s in Fig S1) and compare the effect over sessions and monkeys against baseline while controlling for subject-by-subject differences by modelling monkeys as random effects.

*3. The authors conclude that "while the results here and in previous studies (Lak et al. 2014, PNAS) demonstrate that activity in the dopaminergic midbrain reflects PEs along a one dimensional scale of subjective value, unexpected reward feature information is not tracked." I do not think these conclusions are warranted, and I also don't think this statement reflects a balanced review of the literature. First, Lak et al. never actually tested responses of dopamine neurons when value-neutral expectations about juice type were violated. Second, value-neutral "sensory" prediction errors have recently been identified in the midbrain by other groups. For instance, in rats, the same dopamine neurons that respond to sRPE also respond to errors in reward identity predictions (Takahashi et al. 2017, Neuron). Similar responses to value-neutral identity prediction errors (Boorman et al. 2016 Neuron; Howard & Kahnt 2018, Nature Communications; Suarez et al. 2019, eLife) and low-level sensory prediction errors (Iglesias et al. 2013, Neuron) have previously been described in the human midbrain. Third, there is a real possibility for false negatives here (see point 1), and thus, interpretation of the current null results regarding RRE in the midbrain need to consider the possibility that 2 drops may not have been discriminated reliably from 1 and 3 drops.*

Thank you for pointing out this issue in such a balanced way even though it is clear that our presentation of this issue might have been inappropriate. We did try and discuss some of the studies that the reviewer mentions but we can see that we have not managed this well. In the revised manuscript we have noted all the studies cited and we have refined the way in which the paper by Lak is discussed. We have also attempted to clarify our argument in two important ways. First, we have pointed out that while the rare reward events (RREs) resemble reward identity prediction errors in that they are not reducible to scalar reward prediction errors, we clarify that they are not the same as a reward identity predictions error either. Just because a brain area does or does not respond to a RRE does not mean that it will or will not respond to a reward identity prediction error. Second, we clarify that absence of evidence for a RRE signal in the ventral tegmental area/substantia nigra (VTA/SN) must be interpreted with caution because absence of evidence does not amount to evidence of absence. We clarify that, instead, what can be inferred from the results is that a clear RRE effect is detectable in the posterior orbitofrontal cortex (OFC) while a clear sRPE result is seen in SN/VTA. In the Discussion section of the revised manuscript we included the following paragraphs:

Several other studies have examined whether the activity of dopaminergic neurons or the BOLD signal in the dopaminergic midbrain responds to changes in the type of reward experienced even when its scalar value remains the same [1-4]. These studies have highlighted important similarities in the way in which the dopaminergic midbrain responds to both value and reward identity prediction errors. For example, across participants, there are correlations in the sizes of responses to value and identity prediction errors [1]. The RREs that we studied here are similar in some respects to identity prediction errors. They are both changes in the nature or type of reward rather than its value. However, the identity prediction errors investigated in previous studies reflect changes in features of the reward outcomes that are intrinsic to the outcomes themselves. By contrast, the RREs that we study here are neither changes in reward value nor changes in a taste or odor identity feature intrinsic to the reward itself. Instead RREs are events that are only surprising in the context of knowledge of the composition of the current reward environment. It is only if the monkeys represent this aspect of the task space they are navigating that they will detect the occurrence of RREs.

The absence of a VS response in the current study is also interesting. In theory it is possible that if the dopaminergic midbrain responds to surprising sensory events such as surprising visual stimuli then this could underlie the dopaminergic response to identity prediction errors. It has been reported that the human dopaminergic midbrain is responsive to surprising visual identities in a study employing face and house stimuli [5, 6]. On the other hand, it has been recently reported that reward identity prediction error responses in the dopaminergic midbrain are not proportional to the perceptual dissimilarity between the different rewards [2] suggesting that reward identity prediction errors are computed in some more abstract reward space.

There are three ways in which we might interpret the current failure to find VS responses in the dopaminergic midbrain. One possibility is that the absence of VS responses reflects the difficulty of imaging a small brain region such as the dopaminergic midbrain. From this perspective it is important to remember that absence of evidence for an effect is not tantamount to evidence of absence of the effect. Against such considerations, however, it would also be important to note that VS effects were found in the current study in several cortical regions that correspond with those previously seen to be responsive, in humans, to surprising visual stimulus identities and that our imaging techniques were sensitive enough to identify sRPEs in the dopaminergic midbrain [5, 6]. A second possibility is that there is a fundamental difference between the visuospatial surprise we have studied here and the visual identity surprise studied in previous investigations [5, 6]; maybe the dopaminergic midbrain is responsive to the latter but not the former. Finally, it is possible that human participants in the previous study obtained some pleasure or satisfaction in correctly predicting the visual stimuli they were about to see even though, as in the current study, these were carefully decorrelated from reward events. It has certainly been argued that human participants are often motivated by a desire to perform a task well and are engaged by some of the task’s design features as much or more than by the monetary rewards they will receive at the end of the experiment [7]. From this perspective, it would seem that when this level of task engagement is lacking, as in the monkeys in the current study, no dopaminergic response to VS is observable.

Dopaminergic neurons in the midbrain have been reported to response to salient stimulus events in addition to responding to their reward value and reward value prediction error associated with a stimulus [8]. This has sometimes been described as a response to the “physical impact” of the stimulus or to the stimulus’ capacity for “behavioral activation” as opposed to its precise scalar reward value [9]. In theory the type of VS we investigated here might contribute to a stimulus’ impact and capacity for behavioral activation. However, it also important to note that our experimental design and VS analysis controlled for other basic features of the visual stimuli we studied. VS-related activity was identified by comparing neural responses to visual stimuli of the same brightness, in the same locations, and with the same temporal predictability; only the predictability of the stimulus location differed between surprising and unsurprising VS events. Secondly, we also controlled for the “physical impact” of our visual stimuli in our GLMs by including a constant term at stimulus presentation but did not examine this effect further. As in the study conducted by Lak and colleagues, however, this analysis approach in the current study means that sRPE in the VTA/SN cannot be explained away as being the consequence of visual surprise.

*Reviewer #2: The goal of the manuscript is to determine whether canonical reward brain areas demonstrate similar neural signals for reward prediction errors (PEs) to other PEs, such as rare reward surprise and visual cue novelty that instead provide information regarding attributes of a stimulus rather than reward value. Monkeys performed a simple, but clever task designed to dissociate these three types of PE while undergoing fMRI scanning. The manuscript provides evidence for different areas of the brain responding to variables related to classic, scalar PE (striatum and VTA/SN), visual surprise (PFC), and rare reward (striatum & OFC). The manuscript addresses an important gap in the field regarding reward and stimulus prediction. However, there are details in the paper that need clarification before conclusions can be drawn from this work.*

Thank you for the positive comments and for the helpful suggestions for revising the manuscript. We explain below, point-by-point, how we have revised the manuscript in response to the reviewer’s comments.

*Major concerns*

*1. The current organization the manuscript make pieces of it difficult to follow. For example, the abstract very clearly delineates the goals and the variables that will be presented in the paper, but then that same structure is not recapitulated in the introduction to the same extent. It is not until the fifth paragraph of the intro that the authors introduce why they designed the experiment the way they did. It may be helpful to frame that more concretely sooner in the introduction and then follow that structure throughout the rest of the introduction, and the results. Similarly, at the end of the introduction (and abstract) the idea of "learning" is used without introducing or defining it. There are further comments about learning below.*

The reviewer is very right in pointing out that our Introduction could be better structured. We have revised and re-ordered the Introduction in the manner suggested by the reviewer. The three types of surprise are now introduced in turn and we follow this structure throughout this manuscript. To this end we have also changed the order in which we discuss our findings in the Results section (we now discuss RRE before VS there as well).

For our changes to the manuscript in regard to “learning”, please refer to our response to point 4 below. Our revised introduction is as follows:

**Introduction**

Animals learn from the past to predict the future. This enables them to adjust to their environment and is critical for survival. One type of prediction that animals make concerns the reward value of potential choices that might be taken [10, 11]. After the choice and the outcome is experienced, the correctness of the original expectation is evaluated. When the outcome is better than expected then there is a positive prediction error (PE) and the animal should revise its estimate of the choice’s future value upwards. When the outcome is worse than expected – there is a negative PE – the animal should revise its future estimate of the choice’s value downwards.

Neurophysiological investigations in animals have shown that areas such as the dopaminergic midbrain encode reward PEs [10, 12-16]. In addition, human functional magnetic resonance imaging (fMRI) studies have also examined whether activity throughout the brain reflects reward PEs [17-23]. While some neuroimaging studies have found evidence for PE coding in the dopaminergic midbrain, they have also reported PE coding in other structures including striatum and prefrontal cortex. One aim of the current study was, therefore, to investigate the nature of PE coding in macaque prefrontal cortex and compared it with that seen in other parts of the brain.

We also, however, intended to address a more general debate about the precise nature of the reward expectations that animals hold. While it is clear that they hold representations of average expected reward size, they also hold more specific representations about the nature and identities of the outcomes that they hope to receive after a choice [24-32]. If that is the case then it might be anticipated that they would also experience PEs not just about the amount of reward – scalar reward value -- but about other features of the reward [33].

It is possible that PE-related activity may actually reflect either scalar reward PEs or PEs about other features of the surprising reward experience [34, 35]. In the current study we disentangled two distinct types of reward PEs reflecting the richness of the reward representations held by the monkeys. Specifically, we wanted to look at rare reward event (RRE) signals that go beyond the scalar reward PE (sRPE) typically studied. The frequency at which a reward occurs is important information that should be keenly monitored for learning purposes. Animals may have a representation of the average standard reward to be expected from an average trial to guide their behavior [36]. Pierce and Hall [37], suggested reward novelty is an important driver of the associability of any stimulus.

In the current experiment, it was possible to motivate behavior with primary reinforcers delivered directly to animals’ mouths. Because of the animals’ training with relatively small but discriminable quantities we could vary reward magnitudes parametrically and have a sufficient number of trials for animals to learn and change their reward expectations. To produce RREs it was necessary to dissociate the reward frequency from absolute PEs. Therefore, we inverted the usual experience of receiving rewards close to the mean reward level most frequently. We did this by making actual instances of reward at the mean level very unlikely. This was by achieved by devising schedules delivering mostly either one or three drops but occasionally delivering two drops. In such a schedule the average reward expectation is close to two drops but actual instances of two drops occurred only rarely. In other words the reward distribution was a bimodal distribution with two equal peaks either side of the mean. Thus, while the delivery of a two drop reward would entail no sRPE, because it matched the average reward amount expectation, it should constitute a large RREs because the delivery of two drops is such a rare and novel experience. Careful experimental design ensured that sRPE and RREs shared only 0.049% of variance so that their neural correlates could be dissociated from one another.

However, as the world is full of minor or currently irrelevant changes, it is beneficial to selectively monitor reward frequency only when actively learning. Thus, we also examined whether increasing reward volatility (known to boost learning) [38, 39], also increases attention to other reward features such as RREs. We employed two types of schedules that we refer to as “changing/learnable” and “stable/unlearnable” (Fig 1A). Animals should learn to expect either a higher or lower average reward in different parts of the changing/learnable schedules, potentially rendering a RRE more surprising or noteworthy. By contrast, in the stable/unlearnable sessions, the two primary outcomes remain equally likely throughout so no outcome is more frequent or expected and a RRE should be less surprising too.

In human neuroimaging studies it is often difficult to determine if all PE-related activity reflects encoding of the reward PE *per se*. This is because it is difficult to motivate human subjects to perform tasks in the MRI scanner with primary rewards. Instead task performance is typically motivated by visual cues that act as secondary, or other higher order cues that predict money given to the participant at the end of the experiment. It is therefore possible that apparent reward PE-related activity simply reflects the visual surprise associated with the appearance of a particular visual cue that is acting as a secondary reinforcer rather than the surprising level or nature of the reward it predicts [34, 40]. That visual surprise may be confounded with reward PE is an important consideration because it has been argued that dopaminergic neuron responses reflect the salience of cues and not just reward PEs [41]. While the salience of cues may be affected by many factors such as physical intensity, visuospatial surprise – appearance at a surprising location may contribute to salience. In addition to comparing sRPEs and RREs, we also considered whether reward PE and visuospatial surprise are encoded in the same manner by the same brain structures.

Specifically, we designed our experiment to concurrently measure the impact of visuospatial stimulus surprise (VS) and two distinct types of primary reward PEs. To infer levels of neural activity across the brain and when testing for the existence and distribution of distinct types of error signals in prefrontal cortex and subcortical areas, we used fMRI. By studying macaques, it was possible to examine neural responses to PEs concerning primary reward that were of consequence and interest for the animals instead of visual tokens like those typically used in human neuroimaging. By carefully designing the order in which visual stimuli and rewards were delivered not only did we decorrelate sRPEs and RREs, we also decorrelated both sRPE and RREs from VS. It was therefore possible to identify neural activity associated with each type of surprising events.

*Another organizational item is the order in which the figures are discussed. At several places in the manuscript the panels of the figures are introduced not in the order that they are presented. For example Figure 1B is first referenced in the introduction without having referenced Fig. 1A. In the results, Figure 3B is discussed before Figure 3A. In general, I think it would be helpful to introduce results in the brain as a whole before discussing results in ROIs.*

Again, we have revised the re-ordered the figures in the manner suggested by the reviewer. We have tried to ensure that the order of calls to figure panels in the main text corresponds to the order in which the panels appear in the figures themselves.

However, we have not changed our structure in regard to discussing the whole-brain and ROI results separately. We feel that in order to stick to the same structure throughout the whole manuscript—discussing sRPE, RRE and VS in turn, as suggested in the first point of the reviewer—it is more accessible to the reader to fully discuss each brain region (both in the whole-brain analysis and in the ROI) for each type of surprise before moving on to the next region. Additionally, this way we can visualise the whole-brain results and the ROIs we placed at the peak activity in the same figures instead of displaying ROIs in additional separate figures. We hope that, given all the other changes we made to the structure of the manuscript along the lines suggested by the reviewer, the reviewer agrees with our assessment of this issue.

*2. It would be helpful to have more of the experimental details more up front. For example, the total number of sessions, runs, trials etc. for each trial type in each animal were not apparently reported. What was the error rate of each animal? Given that relative instances of each kind of trial are very important across the manipulations, establishing the actual numbers and frequencies based on the animal's actual performance of each I feel is important. References are made in the results to the average number of trials and sessions, but more specifics would be helpful.*

We have revised the manuscript so that this material appears near the beginning of the Results section. There, we now report how many sessions of each schedule type each monkey completes and their average lapses over all completed sessions. For example, for the stable sessions we now report that

Five animals completed six of these sessions and one animal completed five of these sessions.

Similar sentences for the other session types. We also report that

Our six monkeys lapsed 15.95%, 15.17%, 11.80%, 10.24%, 9.89%, and 6.89% on average over all sessions they completed.

*A second important detail that needs more clarification is the use of the striatum and PFC large VOI. In my first read through of the results, it is was not at first clear how the VOI was being used. I think the manuscript should front this information much more strongly and be careful about the use of the phrase "whole brain". Also, for one analysis, of VS, the manuscript mentions one whole brain alternative analysis that is presented in the supplement (top of pg 16, Sup. Fig 6). In the discussion exploratory analyses showing IPS and IPL are mentioned, but I do not think those analyses were introduced in the results, nor are they shown in the supplement. I very strongly feel that even if the authors want to focus on the frontal cortex and striatum for their main analyses, that that whole brain analyses without the use of the large VOI should be shown, even if it's at lower threshold, in the supplement. It is difficult to contextualize how unique these results may or may not be to these regions without this information.*

The reviewer is raising an important point here and we have revised our manuscript to make our methods and the reasoning behind our methods clearer. Moreover, we note that on re-reading our manuscript that we had indeed incorrectly referred to some analysis as whole brain analyses when in fact they were conducted within a relatively large VOI. We apologise because we realize that this error must have been confusing for the reader. In the revised manuscript we have clarified that we used a VOI in our analysis because we only wanted to discuss and interpret results in frontal regions (and in the basal ganglia and the dopaminergic midbrain) as we had expected that the signal in some of these regions might not survive a spatial cluster-based analysis approach that emphasize effects of greater spatial extent. We explain how the frontal cortex, striatum, and dopaminergic midbrain were the regions we suspected to be involved in the decision-making and learning processes we were investigating.

However, equally importantly, we make clear in the revised manuscript that these initial fears were perhaps too great; even without the VOI, all the main effects we showed in the original manuscript still survive cluster correction (even including those in dopaminergic midbrain) so in fact employing the VOI made no difference to the statistical significance of results. It only produces slight changes to the peak locations of some results in the striatum. When no a priori VOI is used to focus on the striatum then some activation peaks are biased by activity in adjacent structures such as the pallidum.

In the revised manuscript we now report the results of the VOI-based analysis as before but in addition, as requested by the reviewer, we include our full report of the results across the entire brain using a standard cluster-based correction approach. We had already mentioned effects outside of the VOI in the Discussion so again little has changed but now this discussion is contextualized more clearly and related more transparently to tables in the supplementary materials as the reviewer suggested. The revised manuscript now reads as follows:

***FMRI:***

In our experiment, expectations could be violated in three main ways: VS, sRPE, RRE. To identify brain areas associated with these three types of surprising event, we ran a three-level multiple regression analysis by employing a general linear model (GLM). For each monkey we used a fixed-effects model between session and applied the FLAME 1+2 procedure from the FMRIB Software Library (FSL) on the highest hierarchical level (level combining animals). We focus on effects on frontal cortex, striatum, and in the vicinity of the dopaminergic ventral tegmental area and substantia nigra (VTA/SN) in the midbrain because these areas are the ones that have been most frequently related to reward value expectation and prediction error coding in both human and non-human primates [9, 11, 23, 42-46]. For this reason we analyzed data in a volume of interest (VOI) covering frontal cortex and striatum (Supplementary Fig S3), and a precisely localized region of interest (ROI) in the much smaller dopaminergic midbrain region VTA/SN. To further examine the co-occurrence of effects within the VOI, we place functional ROIs at peaks of the sRPE, RRE and VS effects.

In this way we attempted to ensure that we were both able to detect surprise and prediction error responses wherever they occurred in the striatum or prefrontal cortex; such responses have previously been reported in several sub-regions in these structures, moreover within the striatum it is not clear that the strongest prediction error/surprise signals can be mapped onto just the ventral striatum, caudate, or putamen. At the same time, the approach increased the statistical power of our analyses to examine neural activity in brain regions that were of *a priori* interest.

We anticipated that the VTA/SN’s smaller size would preclude other analysis approaches commonly used in fMRI such as spatial cluster-based statistics that are most beneficial when there are large areas of activation, however, the activity in VTA/SN, as in the striatum and frontal cortex was so prominent that it could also be identified using a standard cluster-based correction procedure for multiple comparisons corrected across the whole brain. Thus in addition, whole brain cluster-corrected results are reported in tables S5-S7 and are discussed briefly in the Discussion.

We used data from 71 of the 72 sessions we had acquired (fMRI data from one session were corrupted and unrecoverable). For sRPE, VS, and RRE our hypotheses necessarily focused on activity that was positively related to each type of surprising event but we also note other patterns of activity where they existed.

*A third detail under this heading is the use of other ROIs in the paper. Sometimes they are independently-anatomically defined and sometimes they are defined based on peaks of clusters from the same contrasts they are used to examine. Given the logic presented in the manuscript for using the VOI (the strong a-priori hypotheses about frontal cortex and striatum), it was not clear to me why the authors did not use unbiased-anatomical ROIs throughout. Further, the authors currently group the striatum as a single structure - if anatomical ROIs are used then including CN, Put, NAc etc. and correcting for multiple comparisons may be a more straightforward approach.*

As the reviewer rightfully points out here, we did not properly specify our justification for how we set-up the ROIs in the manuscript. First, throughout the manuscript we used a single anatomical ROI for the SN/VTA, an area for which effects are notoriously hard to find in a cluster-corrected whole-brain analysis because of its size (although as we have noted such effects are present in the current study). This area is known to exhibit a sRPE effect (which we replicate in our manuscript). It was thus of a priori interest for our manuscript to also examine whether this region showed effects of RRE or VS.

However, a priori, it was not possible to know where in the brain might respond to the rare reward events (RREs) that are a major focus in the study. It was therefore necessary to be open to the possibility of seeing effects in a range of other structures. We think that the reviewer’s suggestion of examining activity in predefined caudate, putamen, accumbens subregions of the striatum is a good one but we hope that in the revised manuscript we have explained why using a VOI allows us to identify effects even if they do not correspond to the traditional boundaries between caudate, putamen, and ventral striatum given that we know that these anatomically different regions are not always differentiable functionally using fMRI and that some signals appear only in part of one of these divisions. We hope that by showing supplementary tables in which the whole brain cluster corrected results are presented that we provide the reader with a clear overview of all aspects of our results in the striatum as well as elsewhere.

We note carefully in the revised manuscript that our other approach for identifying activity based ROI’s within our large VOI means that within our initial VOIs we can find locations in which the effects are most prominent. These activity-based ROIs then allow us to investigate the co-occurrence of other effects in an area that we know already encodes one effect. We explain in our manuscript now how, because of this procedure, particular care must be taken to reporting any statistical tests on the ROI to avoid circularity/double dipping. When revising our manuscript we have tried to make clear when the reporting of activity in a functional ROI amounts to a truly independent statistical analysis and when it amounts simply to an illustration of the statistical effect already identified in the initial statistical analysis conducted at the whole brain level or within the VOI. We report such statistics for the ROI selection regressor simply to give an indication of the rough effect size for the reader, but hope it is very clear through the colour scheme, legends and text that those numbers are not meant to be the actual judgement of significance as this is based on the prior whole brain result.

***FMRI:***

In our experiment, expectations could be violated in three main ways: VS, sRPE, RRE. To identify brain areas associated with these three types of surprising event, we ran a three-level multiple regression analysis by employing a general linear model (GLM). For each monkey we used a fixed-effects model between session and applied the FLAME 1+2 procedure from the FMRIB Software Library (FSL) on the highest hierarchical level (level combining animals). We focus on effects on frontal cortex, striatum, and in the vicinity of the dopaminergic ventral tegmental area and substantia nigra (VTA/SN) in the midbrain because these areas are the ones that have been most frequently related to reward value expectation and prediction error coding in both human and non-human primates [9, 11, 23, 42-46]. For this reason we analyzed data in a volume of interest (VOI) covering frontal cortex and striatum (Supplementary Fig S3), and a precisely localized region of interest (ROI) in the much smaller dopaminergic midbrain region VTA/SN. To further examine the co-occurrence of effects within the VOI, we place functional ROIs at peaks of the sRPE, RRE and VS effects.

In this way we attempted to ensure that we were both able to detect surprise and prediction error responses wherever they occurred in the striatum or prefrontal cortex; such responses have previously been reported in several sub-regions in these structures, moreover within the striatum it is not clear that the strongest prediction error/surprise signals can be mapped onto just the ventral striatum, caudate, or putamen. At the same time, the approach increased the statistical power of our analyses to examine neural activity in brain regions that were of *a priori* interest

We anticipated that the VTA/SN’s smaller size would preclude other analysis approaches commonly used in fMRI such as spatial cluster-based statistics that are most beneficial when there are large areas of activation, however, the activity in VTA/SN, as in the striatum and frontal cortex was so prominent that it could also be identified using a standard cluster-based correction procedure for multiple comparisons corrected across the whole brain. Thus in addition, whole brain cluster-corrected results are reported in tables S5-S7 and are discussed briefly in the Discussion.

We used data from 71 of the 72 sessions we had acquired (fMRI data from one session were corrupted and unrecoverable). For sRPE, VS, and RRE our hypotheses necessarily focused on activity that was positively related to each type of surprising event but we also note other patterns of activity where they existed.

*3. There are many places throughout the manuscript where claims are made that one ROI showed responses to one variable while others didn't. What was not clear to me was whether there were statistical comparisons performed to determine if the representations were statistically greater in one area over another.  Are there ANOVAs or similar tests that were performed to show that effects were different? Are there variables that can be directly contrasted against each other? The manuscript mentions one such contrast in Sup. Fig. 6 and perhaps one other, but nothing further. If the manuscript would like to conclude that there are differences in representation of these variables, then it would seem to be appropriate to directly test/contrast them against each other. Also, more comparisons to the equiprobable condition would bolster their claims.*

If we understand the reviewer correctly, they are asking why we are not conducting relative statistical tests that compare activity in one region with activity in another to support our claims of activity in specific brain regions and not others. When it comes to the whole brain analysis this is simply not possible, as there are too many voxels to compare with each other and therefore other methods such as cluster-correction have emerged. However, they only give you an answer about what brain regions appear to have a statistically significant effect and which do not. Once we reduce the number of regions using our functional ROI’s we cannot include such a test as it would not be unbiased because we would be testing activity in a region which we selected based on peak signals against another region that we know is less likely to have the effect to the same degree because we are not selecting its peak signal. This is an issue that is broadly similar to the one that the reviewer cautions us against in the previous point. We could in theory add comparative analyses, which would appear to support our argument, but ultimately those tests would be biased and the statistics would be thus invalid.

The second point of the reviewer is that comparisons with the equiprobable condition would strengthen our claims. The equiprobably condition is designed as a comparison for RRE (but not sRPE or VS), as the frequency of two drops of juice is the key difference between equiprobable and other sessions types. We have carried out contrasts of RRE activity between changing/learnable and equiprobable and between stable and equiprobable sessions and report the results in Supplementary Figure S11. The other possible comparison of these session types is in the anatomical region where we found the RRE effect; this is shown in Supplementary Figure S10. It again identifies a very similar part of the striatum and posterior OFC.

The revised text now reads:

We note that we were able to demonstrate specific prediction error and surprise effects because each region that was identified as showing a significant effect of sRPE, RRE, or VS was then examined to see if it also held either of the two other types of activity patterns. Conducting a test in this way provides a strong demonstration of specificity if the secondary tests fail to find other prediction error/surprise effects even when not correcting for multiple comparisons. We refrained, however, from comparing activity patterns in different areas statistically in case it might be argued that such comparisons are circular given that the areas usually had been first identified by their response in relation to a particular statistical contrast.

*4. I struggled with the design and interpretation of the changing/learnable condition. First, it was not clear exactly what was changing/learnable at first. The manuscript uses "reward rate" I believe to describe that condition, but that would seem to imply that the timing between the rewards would change to be more or less frequent. My interpretation from the methods is that it is not the timing between the rewards that changes, but the relative ratio of one drops or three drops that the monkey receives. Again, this is where trial counts and run structure would be helpful for understanding exactly what the monkeys are doing. A more detailed description of what the manipulation is in the introduction and/or results would be helpful. Was this manipulation in the same session, different sessions, how many?*

We thank the reviewer for pointing out how our usage of the term “reward rate” might be misleading to describe the changing/learnable condition. The reviewer is right, it is not the timing between rewards that changes but the ratio between one and three drops. Throughout the manuscript we changed the word “reward rate” to “average reward” or “mean reward” to avoid any confusion. We have also adjusted panel A in Figure 1 to better reflect the change in average reward in our changing/learnable sessions.

*The manuscript takes careful time to consider what is traditionally thought of as PE and how definitions could be different and how they test those differences. It would be helpful if the manuscript could give the same careful introduction and consideration to possible definitions of learning and what it means in the context of this task that the animals are doing. In some sense, all the conditions are "learnable" because they rely on the animal having a (learned) expectation of the stimuli and the drops of juice. Of course there are many kinds of learning, but often learning is thought of in terms of association, but what is changing in the learnable condition is not an association, but an expectation of the average amount of reward. It was not clear to me on what time scale this change was happening, and if the animal would have any reason to track this information or if there was any behavioral evidence that the animal used this change in information. There is some mention (pg 10) of responses to RREs being different in stable vs. changing, but I did not see further discussion of it.*

*Given all of the above, I felt more details were needed before being able to evaluate the striatum/OFC results for the changing condition. Might it also be true that neural and/or behavioral responses to sRPEs might be different when there are changing expectations?*

The reviewer is correct that it would be beneficial to include a discussion of what learnable means in the context of our task. As the reviewer points out, what is classically thought of as learning in tasks like ours is the associations between stimuli and outcomes. In all our conditions the monkeys can form such expectations, i.e. they can learn the statistics of obtaining one, two, or three drops of juice.

To elaborate on what we mean by changing/learnable, we have changed the relevant panels in Figure 1. There we now show a plot of the estimated average reward from a simulated Rescorla-Wagner learner for all session types. As can be seen visually, in the changing/learnable condition the simulated learner has to re-learn the average reward amount at the beginning of a session (because an average amount of two drops is expected) and halfway through a session when the average reward reverses. In contrast, in the stable and equiprobable sessions the probabilities of 1,2 and 3 drops never change throughout the session and the animal can assume that the average reward amount is close to two drops throughout and does not change dramatically.

Additionally, we have added a paragraph near the beginning of the Results section where we highlight two aspects of this session type that can be learned. It reads

We refer to these sessions as ‘changing/learnable’ in contrast to the ‘stable/unlearnable’ sessions because of two features they have: First, we expect monkeys to have formed strong priors about two drops of juice as the average reward amount because of the high number of stable/unlearnable sessions. Thus, when they encounter a changing/unlearnable session where the reward amount starts at 1.5/2.5 drops, they must re-learn the average reward. Moreover, once the average reward changes halfway through a session, they yet again must re-learn the average reward (Fig 1A bottom right). The second reason we call these sessions ‘changing/learnable’ is that the uncertainty about juice amount (one, two, or three drops) that can be reduced in these sessions via learning is greater than in the stable/unlearnable sessions. This is because the inherent irreducible uncertainty about juice amount that is built into the schedules is greater for the stable/unlearnable than for the changing/learnable sessions: in the stable/unlearnable sessions the monkeys can at best figure out that one and three drops of juice are equally likely (45% and 45%; Fig 1A top) and thus expect them with equal probability. In contrast, in the stable/unlearnable sessions the monkey can figure out that on any trial the ratio between one and three drops is biased (20% and 70%; Fig1A top) and thus form less uncertain expectations about what juice amount to expect.

Because of the simple design of our task we did not find any behavioural evidence that our animals respond differently in the changing/learnable and stable/unlearnable sessions, as the reviewer rightfully pointed out. There is, however, also no clear *a priori* hypothesis about what behavioural difference to expect and we thus are not too concerned about the lack of a result here.

*Other concerns*

*1. There are many details that would be helpful for the clarity of the figures with brains & ROIs on them.*

a. *An indicator of which side of the brain is R/L and the slice numbers for the slices shown I believe is not present in any of the figures.*
b. *The large arrows pointing from the brains to the graphs below make it difficult to understand what volumes/ROIs are being used. For example, in Figure 3A the arrow goes from an area of univariate results where an ROI sphere was used (and it's not clear where the sphere sits in relation to the results shown), whereas in Fig 3B there is no arrow from the anatomical ROI. I would recommend removing the arrows and outlining the area used as an ROI.*
c. *The colored stars to indicate different kinds of significance are confusing.*
d. *The black dashed lines indicating the mean are very very difficult to see*
e. *I appreciate the authors desire to show all the data for each animal, but the many shapes and colors make it difficult to extract the important information from each graph. Perhaps the authors could experiment with other kinds of plots that still show the information (violin plots?) or different colors that show the detail if needed, but it is (visually) easier to background information that is not immediately the most important for each figure.*
f. *If the figures are going to distinguish between stable and changing conditions, then perhaps some analyses as to whether they are the same or different could be provided. It's hard to know if we should take anything from the triangles and squares because they are very hard to see.*

We have revised the figures along the lines suggested by the reviewer. We show an example of a new figure below:

The only thing we did not change as requested by the reviewer is point c above. This is because we feel that is very important for the correct understanding of our analyses that the reader is aware that statistical significance was obtained in two distinct ways. Statistical significance denoted with a red star is according to the whole brain analysis; the plot showing the z-statistics in the corresponding ROI are thus only an illustration of it. This is because we already know that the average z-statistic will be positive. In contrast, the z-statistics for other regressors extracted from the same ROI can still be tested in an unbiased way, which is why we perform these statistical tests and report their results within the figure. We hope that the reviewer agrees that keeping this visual distinction between the different kinds of statistical tests in the figure does justice to our analysis strategy.

*2. There are mislabeled figure references in text for Fig 2. A and B- page 10. With reference to the columns- the order of the graphs is reversed: text states 'sixth column in Fig 2A,
second column in Fig 2B'. (Fig 2A only has 5 columns total)*

Thank you for catching this. We have corrected the calls to the figures so that the text and figures correspond.

*3. There is reference to GLME5 and GLME6 based on RT analysis in the methods, but results and further reference to these are not mentioned with that section (pg. 10) or elsewhere.*

Again, thank you for catching this. We have included a call to the relevant supplementary figure where GLME5 and GLME6 are mentioned in the text.

*Reviewer #3: This manuscript describes an fMRI experiment in rhesus macaques that examines the neural correlates of scalar reward prediction errors, visual surprise, and rare reward events. The latter to potentially account for how surprise might drive learning based on perceptual versus reward inference processes, and constitute the main conceptual advance of the manuscript. BOLD activity in the putative dopaminergic midbrain and striatum was correlated with RPEs (only in the left VTA) and not VS or rare reward events. Activation in lOFC was correlated with visual surprise (i.e. whether a cue associated with different amounts of reward appeared in a frequent versus rare location). Posterior lOFC was identified as encoding rare reward events, but in only in sessions where the mean reward rate drifted up or down over the course of the session. While interesting, several aspects of the experimental design and analyses raise concerns as to the reproducibility and robustness of the reported results.*

Thank you for your comments on our manuscript. We hope that our replies below address your concerns.

*Fitting a value updating algorithm to the beta coefficients resulting from a general linear model fit to predict performance lapses (i.e. GLME2) to derive learning rates and sRPEs is a circular analysis strategy, because GLME2 already accounts for value integration by including the reward outcomes on the previous five trials. Instead the value updating algorithm should be used to estimate value updates directly with free the learning rate set as a free parameter to either predict performance lapses or reaction times. The authors could even use arbitrary learning rates, as it was recently demonstrated that model-fitting isn't necessary for identification of model-based fMRI results (Wilson & Niv, 2015). Do the authors obtain similar results when they estimate sRPEs directly based on the empirical reward value and an arbitrary learning rate?*

We thank the reviewer for these suggestions. This paragraph points out two potential issues with our analysis, which are (1) a circularity in our approach to derive learning rates and (2) the robustness of our analysis if other (arbitrary) learning rates are used. We will answer both points in turn.

First, the analysis we performed is not circular. We do, however, acknowledge that a lack of details in the first draft of our manuscript may have allowed the reviewer to form this impression and, in retrospect, we realize that it may have been easy to misunderstand what we actually did. We apologize for the lack of detail and will provide an explanation for why we chose to analyse the data in the way we did below.

A classic Rescorla-Wagner reinforcement learning model updates the reward expectation $Q_{t}$ based on the reward history $Q_{t-1}$ and the newly received reward $r_{t-1}$ according to the formula

$Q_{t} = \alpha r_{t-1}+(1-\alpha)Q_{t-1}$,

where $\alpha$ is the learning rate. This formula can be expanded by substituting $Q_{t-1}$ which is $Q_{t-1} = \alpha r_{t-2}+(1-\alpha)Q_{t-2}$, into the equation, such that

$Q_{t} = \alpha r_{t-1}+(1-\alpha)(\alpha r_{t-2}+(1-\alpha)Q_{t-2})$.

By repeatedly substituting $Q_{t-2}, Q_{t-3},$ etc. in this manner and rearranging terms, it can be shown that

$Q_{t} = \sum_{\tau=1} \alpha(1-\alpha)^{\tau-1}r_{t-\tau}$.

To generate choice probabilities from this reward expectation, Q_t_ is substituted into a sigmoid function

$$p_{t} = \frac{1}{1+e^{-\beta Q_{t}}}$$

and multiplied with the inverse temperature $\beta$. In our task this probability corresponds to the probability of not selecting the stimulus (a “performance lapse”), while (1-p) is the probability of selecting the stimulus. The reinforcement learning model we are fitting to our data is mathematically equivalent to fitting a truncated reinforcement learner. Unlike a full reinforcement learning model, which calculates the current expected reward based on the full reward history, such a truncated model only takes into account the reward history from the past *n* trials (the *n* we are using in our analysis is *n=5*), i.e.

$Q_{t} = \sum_{\tau=1}^{n=5} \alpha(1-\alpha)^{\tau-1}r_{t-\tau}$.

Before we discuss the reasons for using such a truncated model, we will show how our analysis is equivalent to it (and thus not circular). In the manuscript we use logistic regression to predict performance lapse on the current trial from the rewards of the previous 5 trials, i.e. $r_{t-1} to r_{t-5}$. Mathematically, fitting this logistic regression can be thought of as finding the coefficients $b_{1} to b_{5}$ that best describe the probability $\pi$ of an attentional lapse occurring on a trial, i.e.

$\pi_{t} = \frac{1}{1+e^{-\sum_{\tau}^{n} b_{\tau}r_{t-\tau}}}$.

As can be seen from comparing the formulas for $p_{t}$ and $\pi_{t}$, they are nearly identical. In fact, by substituting the value of $Q_{t}$ of a truncated reinforcement learner into $p_{t}$, we obtain

$p_{t} = \frac{1}{1+e^{-\beta\sum_{\tau=1}^{n=5} \alpha(1-\alpha)^{\tau-1}r_{t-\tau}}}$.

Thus, if the observed behaviour is generated by a truncated reinforcement learner, then the optimal coefficients of the logistic regression should be

$b_{\tau}= \beta\alpha(1-\alpha)^{\tau-1}$, for $\tau= 1,...,5$.

We can use this fact to estimate both the learning rate and the inverse temperature from the coefficients of the logistic regression by finding the $\alpha$ and $\beta$ that minimize the sum of the squared difference between $b_{\tau}$ and $\beta\alpha(1-\alpha)^{\tau-1}$ for all $\tau.$ We have thus described the five coefficients as an exponential kernel with two free parameters $\alpha$ and $\beta$ (which is why we use two degrees of freedom when testing for its significance in the main text).

The choice of truncating our reinforcement learner at *n=5* was made because estimating the coefficients that are associated with more distant rewards is less reliable (which is not accounted for when fitting $\alpha$ and $\beta$ this way). With an estimated learning rate of $\alpha=0.257$ for our study the term $\sum_{\tau=1}^{n=5} \alpha(1-\alpha)^{\tau-1}=0.7736$. This means that 22.64% of the influence that reward history would have for a regular Rescorla-Wagner learner does not affect our truncated learner.

The reason we used this method in the manuscript is that it allowed us to also include additional terms in the logistic regression, such as whether a rare reward event or a visuospatial surprise occurred. We can thus estimate the learning rate while controlling for other experimental factors. Controlling for other factors is problematic if the learning rate is estimated using the traditional method of fitting a reinforcement learning model but controlling for these factors is important.

In the revised manuscript we have rewritten the part of our Results section related to this analysis. It now reads:

To estimate the learning rate we used to calculate the sRE, we used another GLME (GLME2, Fig 2B) that included the reward on the previous five trials as individual regressors, and then estimated a Rescorla-Wagner type reinforcement learning model from these beta weights by finding the Rescorla-Wagner model that best describes the observed weights on previous rewards. We thus obtained a learning rate (alpha = 0.257). This procedure is broadly equivalent to fitting a truncated reinforcement learning model which also controls for other confounds (see Materials and Methods for details).

In the Materials and Methods section we now further elaborate on this:

The learning rate to compute sRE in GLME1 was obtained from GLME2. This was done by minimizing the Euclidean norm between the weights of the previous five rewards of a truncated Rescorla-Wagner reinforcement learning model (described by two free parameters: a learning rate and an inverse temperature) and the regression coefficients for Rewardt-1 to Rewardt-5 in GLME2. This procedure allowed us to both fit a learning rate while simultaneously accounting for other experimental factors in the linear model.

Nevertheless, we agree with the second point of the reviewer that it is worth checking what difference it makes if we calculate the learning rate the traditional way (or any other arbitrary way). When we calculate the learning rate the traditional way, we obtain an estimate of 0.280, whereas the way of estimating it as described above yielded 0.257. As explained above, these differences are because the second learning rate comes from a truncated reinforcement learner and controls for other experimental factors but it is reassuring to see that the two values are within the same range.

Below we test the robustness of our results if we calculate the prediction errors with a range of learning rates. We have done this by focussing on the VTA/SN ROIs (averaged over both hemispheres) as discussed in the main manuscript. We extracted the BOLD timecourse from this region and then ran a linear regression on each timepoint. Our GLM contained a constant term, a regressor coding for VS, a regressor coding for RRE, the trial number, and a regressor coding for sRPE. The effect size of a regressor was determined by averaging over a 10s window after reward delivery. By varying the learning rate with which we computed the sRPE regressor we were able to determine the effect size associated with different learning rates, as shown below

The red line indicates our behavioural learning rate of 0.257 while the black line is the strength of the sRPE effect in the timecourse for different learning rates. As can be seen, in the VTA/SN our fitted learning rate is close to the peak of the effect size, indicating a good association between the neural and behavioural data. However, notably the effect is only slightly weaker for other (lower) learning rates as indicated by (Wilson & Niv, 2015)*.*

We have included this new figure in the supplementary materials for the revised manuscript. The main text now states:

Finally, we considered the possibility that the sRPE effect might simply be artefact of the learning rate used when estimating monkeys’ reward value expectations. In line with observations previously made by Wilson and Niv [47] a control analysis provided evidence for sRPEs in the dopaminergic midbrain and striatum regardless of the precise learning rate used (Figure S7).

The legend for the supplementary figure we added reads:

To consider the possibility that sRPE signals might be an artefact of the learning rate we used when estimating the monkeys’ reward value expectations, we examined the BOLD response in the VTA/SN ROI further. By running a linear regression on the extracted BOLD timecourse time-locked to reward delivery (with terms coding for a constant, sRPE, VS, RRE, and trial number) we can determine the effect size of the sRPE regressor when constructing it using different learning rates. Effect size is here defined as the averaged beta weights for regressions run on the 10s after reward delivery. The red line indicates the learning rate we used in the main text (0.257). As can be seen, the empirical learning rate is close to the peak effect strength in VTA/SN but the effect strength is positive throughout regardless of learning rate.

*For the ROI analyses were scalar RPEs, visual surprise, and rare reward event regressors tested separately or in the context of the same model? If they were not assessed simultaneously then a correction for multiple comparisons needs to be applied.*

In brief, the answer is yes, these regressors are tested simultaneously in the same model.

One might expand on this answer, however, and note that what the reviewer points out here relates to a bigger issue in the analysis of functional MRI data and indeed other GLM-based analyses in other fields. The standard way to analyse such data on the whole brain level (as in many other fields), and the approach which we also employed here, is to include all relevant factors into the same linear model that is fitted on BOLD activity. As such, the various regressors (sRPEs, VS, RRE and other confounds) compete for variance in the dependent variable – the BOLD signal. To then examine statistical significance, contrasts are applied that specify which (combination of) regressors should be compared to each other or to baseline. The standard approach is not to apply any penalty on the p-values for the number of different contrasts used at this stage but to only apply any further (cluster-level) corrections of p-values within each contrast (given the number of simultaneous tests performed across voxels – consideration is only given to spatial clusters of voxels in which the same effect is found across adjacent voxels). Here, we employ the same analysis strategy for our ROIs: we extracted the z-statistics for sRPE, VS and RRE that were fitted together in the same model but then separately tested the fitted values of each against zero without penalizing the p-values. Our approach is thus very much in line with the standard practice in the field that is implemented in all major toolboxes for fMRI analysis. As such, we left the p-values as they are in the manuscript and did not adjust them.

More generally, when we test within an ROI we were interested in whether its signal is selective for one factor or whether there is any evidence it also co-localizes other types of prediction errors. Thus, we were interested in selectivity and the harshest form of statistical testing is to NOT apply multiple comparison correction to see whether there is any evidence for an additional other prediction error signal (e.g. sRPE, VS or RRE). In other words, a second stage in our analysis strategy was actually to test formally the selectivity of coding in each area. This is done by, for example, testing whether an area found to encode sRPE at the whole brain level also codes for VS, or by testing whether an area found to encode RRE at the whole brain level also encodes sRPE. Again, the harshest and most exacting test of selectivity here is, therefore, not to apply any penalty for multiple comparisons when conducting these additional statistical tests. This is because testing for any other effects in the same ROI without penalizing these tests gives the highest chance to discover other effects in the area. If we were to penalize these other tests then it could be argued that we are unfairly setting ourselves up to conclude there is selectivity because any other effect in our ROIs would have to be quite substantial to still come out significant after correction for multiple comparisons. In summary, activity in several areas is selectively related to specific types of surprise despite careful testing for the possibility that other types of surprise drive activity in the same area.

The reviewer is very right in pointing out that we should have clarified this. In the revised manuscript, we make clear that the scalar RPEs, visual surprise, and rare reward events were fitted simultaneously in the same model by clarifying each time we mention that we extract z-statistics that they are obtained from the same whole-brain analysis. For example:

Extracting the z-statistics of each session from the individual regressors of our whole-brain analysis from spherical regions of interest (ROIs) with a 10mm diameter in the left and right striatum respectively confirmed a clear effect of sRPE (Fig 3C).

Similar text inserts were added throughout the manuscript.

*Dopamine neurons have been shown to code both visual novelty responses as well as reward prediction errors (Lak et al., 2016). Their initial phasic activity reflects novelty while a later component of their activity encodes RPEs related to learning. It is therefore surprising (or maybe not given the low SNR of BOLD activity in this region) that the authors would not find neural correlates of visual surprise in their VTA/SN analyses.*

The visual novelty responses that the reviewer refers to in the work of Lak, Stauffer, Schultz (eLife, 2016 and reviewed by the same authors in Current Opinion in Neurobiology; Schultz et al., 2017) the authors sometimes refer to as “physical impact” of the stimulus. This phrasing nicely makes clear that the dopaminergic neuron response can be considered to comprise two components. The first component is a transient response to the occurrence of the onset of the reward predicting stimulus and the second component scales with the expected reward value associated with the stimulus and reward prediction error. Exactly the same is true of the BOLD signal we record from the same region here. To clarify, our VS signal is the degree to which stimulus processing is affected by prior expectation of the physical location of the stimulus, not the physical impact itself. We clarify in the revised manuscript that in our GLM a term that we refer to as the “decision constant” captures variance in the BOLD signal that is related to the occurrence of the stimulus. This part of the BOLD signal, capturing the physical occurrence of the signal, resembles the first component of the dopaminergic response, reflecting “physical impact”, recorded by Lak, Stauffer, and Lam. Variance in the BOLD signal in the dopaminergic midbrain is also significantly related to scalar reward prediction error and this approximately corresponds to the second component of the dopaminergic response described by Lak, Stauffer, and Lam. Because, like Lak, Stauffer, and Schultz, we have relatively little to say about the decision constant effect / physical impact effect we have limited the degree to which we have discussed this issue even in the revised manuscript.

While these correspondences between our results and those reported by Lak, Stauffer, and Schultz are clear, it is equally important to realise that the manipulation of visual surprise we carry out is quite different to any manipulation reported by Lak, Stauffer, and Schultz. When we examine visuospatial surprise, we are examining the activity in the dopaminergic midbrain in relation to the spatial position of the stimulus in order to test whether activity changes when the stimulus is in an unsurprising location (a location that it has just appeared in) or a surprising location (a location that it has not just appeared in). The analysis controls for the variables that Lak, Stauffer, and Schultz were interested in – the “physical impact” of the stimulus – because both surprising and unsurprising visual stimuli are equal in physical intensity and are equal in their temporal predictability.

In our revised manuscript we clarify how our results replicate the findings of Lak, Stauffer, and Schultz in finding that there is a scalar reward prediction error-related response in the dopaminergic midbrain even after controlling for the “physical impact” of the stimulus but that the “physical impact” of the stimulus also drives a response. We clarify how visual surprise, in the sense in which it is used here, is unrelated to any factor considered by Lak, Stauffer, and Schultz. In the Materials and Methods sentence, we have rewritten the section related to this regressor to further clarify its role:

Finally, we also included two constants, one at decision and one at outcome, as well as a regressor that controlled for hand movements registered by the infra-red touch sensor into the GLM. The decision constant accounts for the neural impact of stimulus presentation. The outcome constant accounts for the neural response to the occurrence of any reward event, regardless of magnitude.

We also clarified in the abstract what we mean by visuospatial surprise by writing:

Additionally, the stimulus sometimes appeared in a surprising location throughout all sessions.

In the results we make this point clear as follows:

It is perhaps worth noting here while the aspect of VS we consider here might contribute to the salience of a stimulus so will other factors. While no VS effect was found in the vicinity of the dopaminergic midbrain, it is quite possible that other aspects of visual stimuli contribute to the transient response that has been noted to occur to the onset of a visual stimulus associated with reward. Schultz, Lak, and Stauffer [8, 9]refer to this response to the onset of a reward-predicting stimulus as being due to the “physical impact” of the stimulus. By contrast, the VS effect we examined compared the neural response to identical stimuli that were equally unexpected in time but which differed because they were either in an unexpected or expected spatial location.

In the Discussion section we now also cite the two papers discussed above and how they relate to our findings:

Dopaminergic neurons in the midbrain have been reported to response to salient stimulus events in addition to responding to their reward value and reward value prediction error associated with a stimulus [8]. This has sometimes been described as a response to the “physical impact” of the stimulus or to the stimulus’ capacity for “behavioral activation” as opposed to its precise scalar reward value [9]. In theory the type of VS we investigated here might contribute to a stimulus’ impact and capacity for behavioral activation. However, it also important to note that our experimental design and VS analysis controlled for other basic features of the visual stimuli we studied. VS-related activity was identified by comparing neural responses to visual stimuli of the same brightness, in the same locations, and with the same temporal predictability; only the predictability of the stimulus location differed between surprising and unsurprising VS events. Secondly, we also controlled for the “physical impact” of our visual stimuli in our GLMs by including a constant term at stimulus presentation but did not examine this effect further. As in the study conducted by Lak and colleagues, however, this analysis approach in the current study means that sRPE in the VTA/SN cannot be explained away as being the consequence of visual surprise.

*Of major concern is that the rare, rewarding event correlate in posterior lOFC only appears in the sessions in which the average reward rate drifts upwards or downwards. As a result of this manipulation the rare, rewarding events delivering 2 rewards now are both surprising but also elicit a prediction error. To accurately identify this region as encoding the rarity of this event versus RPE coding it is necessary to include these RPEs, no matter how small, in the GLM in addition to the rare reward regressor. Otherwise this is a fundamental flaw in the experimental design. Moreover, the lOFC region identified as a neural correlate of visual surprise and rare reward events appears to be driven by two individual monkeys in each case. The error bars for most of the animals contain 0 indicating the effect is not consistent across the animals. Compare this to the scalar RPE correlates observed bilaterally in the striatum, where the error bars for the majority of the animals do not contain 0.*

In this paragraph the reviewer points out two worries with our plOFC result: (1) the rare reward events in the drift sessions are also prediction errors and (2) the reviewer argues that it appears that our effect is only driven by 2 of our 6 monkeys. We thank the reviewer for pointing out both of these worries, which we think are due to us not making clear enough what kind of analyses we are running in the manuscript.

To address the first point, we do, in fact, include the RPEs in our GLM. Thus, the rare reward event effects that we describe were found after controlling for “these RPEs, no matter how small, in the GLM in addition to the rare reward regressor” in precisely the manner that the reviewer prescribes. The full GLM we run for our whole brain analysis included all our regressors of interest, which are scalar RPEs, rare reward evens, and visuospatial surprise, as well as some other additional nuisance regressors (such as image motion, side of response, etc). We describe how we run the whole brain analysis at the very end of the Methods section, which we acknowledge is the least prominent part of the manuscript. We therefore changed the text in the results section to clarify how we are running our GLMs. The text now reads

Note that by including terms relating to both sRPE and RRE in the same GLMs we can ensure that activity actually related to sRPE cannot be misinterpreted as activity related to a RRE.

The second point the reviewer made in this paragraph is about how many of the error bars for our plOFC effects “contain 0”. The reviewer mentions that this might be a problem for a visuospatial surprise effect in the plOFC but we report no such effect in the manuscript; we report an effect of rare reward events in the plOFC not an effect of visuospatial surprise.

The reviewer is also concerned that only in a subset of animals the standard error touches the 0 line (2 out of 6); importantly in 5 out of 6 animals the direction of the effect is in the right direction, which leaves only one animal without evidence for an effect. However, more importantly than such rules of thumb, we wanted to make a statistically rigorous statement about our group of monkeys overall. Therefore it is important to look at the data of the group as a whole. In fact, running the experiment with 6 monkeys (rather than the commonly used 2 or 3) allowed us to compute group statistics over all 6 animals (which we modelled as random effects). While showing statistical effects in pairs of individual animals in neurophysiological experiments is common practice, it is important to remember that statistical analyses on a group level is equally valid and, in fact, this is the more common way in which statistical analyses are conducted beyond the field of non-human primate research.

Perhaps another way to think about this is that the unit of analysis is the individual monkey and the key determinant of statistical significance is in the mean and standard error of the group effect.

What is true, however, is that when research results are reported in this manner, only the mean performance levels for each individual are reported together with the mean and standard error for the estimate of the group mean because this has the simplest and most direct impact on statistical significance. From this perspective, the way the data we present here should be viewed is by noting that for 5 of our 6 monkeys the effect is positive (and the 1 monkey with a negative effect has the smallest effect size). This consistency in the direction of the effect across individuals is what makes the overall group statistical effect significant, as reported in the manuscript. However, to pre-empt misunderstanding by readers, we have revised the manuscript by changing the way in which we display our results (also in line with a request from reviewer #2) to now not show any error bars for individual animals any longer (which we did not perform any statistical tests on). Instead, we now display the group-level standard error, which is the relevant error bar for our statistical test. While all the same data is still present in the figures (and an interested reader can still calculate the standard error for each animal by hand) we hope that this more parsimonious way of plotting the data helps guide the reader towards the relevant statistics.

*"Another way to test for effects of RRE is to examine if activity during changeable/learnable sessions is significantly different from activity during equiprobable sessions." This would also include a key comparison of whether the RRE effect in sessions in which the mean reward rate changes over time differs from sessions in which it remains stable, where an RRE was not detected in posterior lOFC. For Fig. 5 and Fig. S5 the authors should report on direct comparisons of these two session types, as well as comparisons to the equiprobable sessions. Also, it is curious as to why the voxel-wise comparisons of the changing and equiprobable sessions session type fails to identify the same posterior lOFC cluster as shown in Fig. 5.*

We hope that we have now clarified in our previous response that we had already employed what might be called the ‘first way’ to analyse the data that was approved by the reviewer. However, the reviewer is right in pointing out that a comparison between changing/learnable and stable sessions would indeed be of interest. We have added a new supplementary figure S11 to the manuscript that is also shown below:

This figure shows the RRE effect in plOFC in the changing/learnable session and compares its location to the effect when we contrast changing/learnable and equiprobable as well as changing/learnable and stable conditions. As can be seen, there is some activity in plOFC for all three contrasts, but it is strongest if we only plot the changing/learnable sessions. We believe the reason for this is because a null effect in equiprobable and stable sessions will lead to a noisy baseline against which the effect in changing/learnable sessions is compared, which makes it look weaker. Both green and blue clusters are shown without cluster correction as we wanted to illustrate the broad similarity in location, despite the comparison making the signal clusters smaller.

The figure legend for the new supplementary figure reads:

**Fig. S11**. RRE activity in plOFC in changing/learnable sessions (red) is located in the same area as activity when we contrast changing/learnable and equiprobable sessions (blue) or changing/learnable and stable sessions (green). All effects shown here are at a threshold of 2 and are shown without applying any cluster correction.

In the main manuscript we call the supplementary figure and write

Another way to test for effects of RRE is to examine if activity during changeable/learnable sessions is significantly different from activity during equiprobable sessions. Running such an analysis on the whole brain level revealed similar patterns of activity in the striatum and plOFC (supplementary Fig S10; supplementary Fig S11). In particular, our plOFC cluster during changing/learnable sessions remains broadly similar when contrasting RRE effects with the other two conditions separately (equiprobable but also changing/learnable and stable sessions (supplementary Fig S11)).

*The described effects are difficult to evaluate by the symbol and color coding scheme. The plotting conventions adopted in the Supplementary Figures, where the Z-statistics are broken out by session type, should be used in the main manuscript figures. It would also be helpful given that this is a within-subject design to link the plotted statistics across the sessions for each animal.*

We have clarified the presentation of the results in the graphs on the basis of the advice we have received from all three reviewers. Since we do not report any differences by session type for sRPE and VS we have opted for not plotting the different session types separately for these analyses in the main text (in line with what reviewer #2 suggested). The plots showing results split-up by session types can still be found in the supplements for any interested reader, which we hope is a good compromise. For RRE where we do report a difference by session type, the plot in the main text is split up.

*The use of the mask for cluster correction as depicted in Fig. S3 is not well justified and an unconventional practice. Usually such masks correspond to smaller regions of interest (i.e. the VTA/SN) not entire swaths of the brain. Do the identified cortical and striatum clusters not survive more traditional whole-brain cluster correction? Is there some reason that this mask needs to be applied. For example, is the SNR highest in these brain regions?*

The reviewer is raising an important point here and we have revised our manuscript to make our methods and the reasoning behind our methods clearer. Moreover, we note that on re-reading our manuscript that we had indeed incorrectly referred to some analysis as whole brain analyses when in fact they were conducted within a relatively large VOI. We apologise because we realize that this error must have been confusing for the reader. In the revised manuscript we have clarified that we used a VOI in our analysis because we only wanted to discuss and interpret results in frontal regions (and in the basal ganglia and the dopaminergic midbrain) as we had expected that the signal in some of these regions might not survive a spatial cluster-based analysis approach that emphasize effects of greater spatial extent. We explain how the frontal cortex, striatum, and dopaminergic midbrain were the regions we suspected to be involved in the decision-making and learning processes we were investigating.

However, equally importantly, we make clear in the revised manuscript that these initial fears were perhaps too great; even without the VOI, all the main effects we showed in the original manuscript still survive cluster correction (even including those in dopaminergic midbrain) so in fact employing the VOI made no difference to the statistical significance of results. It only produces slight changes to the peak locations of some results in the striatum. When no a priori VOI is used to focus on the striatum then some activation peaks are biased by activity in adjacent structures such as the pallidum.

In the revised manuscript we now report the results of the VOI-based analysis as before but in addition, as requested by the reviewer, we include our full report of the results across the entire brain using a standard cluster-based correction approach. We had already mentioned effects outside of the VOI in the Discussion so again little has changed but now this discussion is contextualized more clearly and related more transparently to tables in the supplementary materials as the reviewer suggested. The revised manuscript now reads as follows:

***FMRI:***

In our experiment, expectations could be violated in three main ways: VS, sRPE, RRE. To identify brain areas associated with these three types of surprising event, we ran a three-level multiple regression analysis by employing a general linear model (GLM). For each monkey we used a fixed-effects model between session and applied the FLAME 1+2 procedure from the FMRIB Software Library (FSL) on the highest hierarchical level (level combining animals). We focus on effects on frontal cortex, striatum, and in the vicinity of the dopaminergic ventral tegmental area and substantia nigra (VTA/SN) in the midbrain because these areas are the ones that have been most frequently related to reward value expectation and prediction error coding in both human and non-human primates [9, 11, 23, 42-46]. For this reason we analyzed data in a volume of interest (VOI) covering frontal cortex and striatum (Supplementary Fig S3), and a precisely localized region of interest (ROI) in the much smaller dopaminergic midbrain region VTA/SN. To further examine the co-occurrence of effects within the VOI, we place functional ROIs at peaks of the sRPE, RRE and VS effects.

In this way we attempted to ensure that we were both able to detect surprise and prediction error responses wherever they occurred in the striatum or prefrontal cortex; such responses have previously been reported in several sub-regions in these structures, moreover within the striatum it is not clear that the strongest prediction error/surprise signals can be mapped onto just the ventral striatum, caudate, or putamen. At the same time, the approach increased the statistical power of our analyses to examine neural activity in brain regions that were of *a priori* interest

We anticipated that the VTA/SN’s smaller size would preclude other analysis approaches commonly used in fMRI such as spatial cluster-based statistics that are most beneficial when there are large areas of activation, however, the activity in VTA/SN, as in the striatum and frontal cortex was so prominent that it could also be identified using a standard cluster-based correction procedure for multiple comparisons corrected across the whole brain. Thus, in addition, whole brain cluster-corrected results are reported in tables S5-S7 and are discussed briefly in the Discussion.

We used data from 71 of the 72 sessions we had acquired (fMRI data from one session were corrupted and unrecoverable). For sRPE, VS, and RRE our hypotheses necessarily focused on activity that was positively related to each type of surprising event but we also note other patterns of activity where they existed.

*While Figure 6 is a nice illustration that might be suitable as a graphical abstract it does not belong as a main figure.*

Whether to include summary figures is normally a matter of taste or editorial judgement. However, as it did not seem to have the hoped clarifying effect, we have removed this figure from the manuscript, as requested, and moved it to the supplementary materials

**References for Reply to Reviewers** (note that number ordering of references differs between this reply and the main manuscript)**.**

1.	Howard JD, Kahnt T. Identity prediction errors in the human midbrain update reward-identity expectations in the orbitofrontal cortex. Nature communications. 2018;9(1):1611. doi: 10.1038/s41467-018-04055-5. PubMed PMID: 29686225; PubMed Central PMCID: PMCPMC5913228.

2.	Suarez JA, Howard JD, Schoenbaum G, Kahnt T. Sensory prediction errors in the human midbrain signal identity violations independent of perceptual distance. eLife. 2019;8. Epub 2019/04/06. doi: 10.7554/eLife.43962. PubMed PMID: 30950792; PubMed Central PMCID: PMCPMC6450666.

3.	Takahashi YK, Batchelor HM, Liu B, Khanna A, Morales M, Schoenbaum G. Dopamine Neurons Respond to Errors in the Prediction of Sensory Features of Expected Rewards. Neuron. 2017;95(6):1395-405 e3. Epub 2017/09/15. doi: 10.1016/j.neuron.2017.08.025. PubMed PMID: 28910622; PubMed Central PMCID: PMCPMC5658021.

4.	Boorman ED, Rajendran VG, O'Reilly JX, Behrens TE. Two Anatomically and Computationally Distinct Learning Signals Predict Changes to Stimulus-Outcome Associations in Hippocampus. Neuron. 2016;89(6):1343-54. doi: 10.1016/j.neuron.2016.02.014. PubMed PMID: 26948895; PubMed Central PMCID: PMCPMC4819449.

5.	Iglesias S, Mathys C, Brodersen KH, Kasper L, Piccirelli M, den Ouden HE, et al. Hierarchical prediction errors in midbrain and basal forebrain during sensory learning. Neuron. 2013;80(2):519-30. Epub 2013/10/22. doi: 10.1016/j.neuron.2013.09.009. PubMed PMID: 24139048.

6.	Iglesias S, Mathys C, Brodersen KH, Kasper L, Piccirelli M, den Ouden HEM, et al. Hierarchical Prediction Errors in Midbrain and Basal Forebrain during Sensory Learning. Neuron. 2019;101(6):1196-201. Epub 2019/03/22. doi: 10.1016/j.neuron.2019.03.001. PubMed PMID: 30897360.

7.	Murayama K, Matsumoto M, Izuma K, Matsumoto K. Neural basis of the undermining effect of monetary reward on intrinsic motivation. Proc Natl Acad Sci U S A. 2010. Epub 2010/11/17. doi: 1013305107 [pii]10.1073/pnas.1013305107. PubMed PMID: 21078974.

8.	Lak A, Stauffer WR, Schultz W. Dopamine neurons learn relative chosen value from probabilistic rewards. eLife. 2016;5. doi: 10.7554/eLife.18044. PubMed PMID: 27787196; PubMed Central PMCID: PMCPMC5116238.

9.	Schultz W, Stauffer WR, Lak A. The phasic dopamine signal maturing: from reward via behavioural activation to formal economic utility. Curr Opin Neurobiol. 2017;43:139-48. Epub 2017/04/10. doi: 10.1016/j.conb.2017.03.013. PubMed PMID: 28390863.

10.	Schultz W. Behavioral theories and the neurophysiology of reward. Annu Rev Psychol. 2006;57:87-115. PubMed PMID: 16318590.

11.	Schultz W. Updating dopamine reward signals. Curr Opin Neurobiol. 2013;23(2):229-38. doi: 10.1016/j.conb.2012.11.012. PubMed PMID: 23267662; PubMed Central PMCID: PMCPMC3866681.

12.	Schultz W, Dayan P, Montague PR. A neural substrate of prediction and reward. Science. 1997;275(5306):1593-9. Epub 1997/03/14. PubMed PMID: 9054347.

13.	Tobler PN, Fiorillo CD, Schultz W. Adaptive coding of reward value by dopamine neurons. Science. 2005;307(5715):1642-5. PubMed PMID: 15761155.

14.	Stauffer WR, Lak A, Yang A, Borel M, Paulsen O, Boyden ES, et al. Dopamine Neuron-Specific Optogenetic Stimulation in Rhesus Macaques. Cell. 2016;166(6):1564-71 e6. doi: 10.1016/j.cell.2016.08.024. PubMed PMID: 27610576; PubMed Central PMCID: PMCPMC5018252.

15.	Chang CY, Esber GR, Marrero-Garcia Y, Yau HJ, Bonci A, Schoenbaum G. Brief optogenetic inhibition of dopamine neurons mimics endogenous negative reward prediction errors. Nat Neurosci. 2016;19(1):111-6. doi: 10.1038/nn.4191. PubMed PMID: 26642092; PubMed Central PMCID: PMCPMC4696902.

16.	Hart AS, Rutledge RB, Glimcher PW, Phillips PE. Phasic dopamine release in the rat nucleus accumbens symmetrically encodes a reward prediction error term. J Neurosci. 2014;34(3):698-704. doi: 10.1523/JNEUROSCI.2489-13.2014. PubMed PMID: 24431428; PubMed Central PMCID: PMC3891951.

17.	O'Doherty JP, Dayan P, Friston K, Critchley H, Dolan RJ. Temporal difference models and reward-related learning in the human brain. Neuron. 2003;38(2):329-37. PubMed PMID: 12718865.

18.	O'Doherty J, Dayan P, Schultz J, Deichmann R, Friston K, Dolan RJ. Dissociable roles of ventral and dorsal striatum in instrumental conditioning. Science. 2004;304(5669):452-4. PubMed PMID: 15087550.

19.	Hare TA, O'Doherty J, Camerer CF, Schultz W, Rangel A. Dissociating the role of the orbitofrontal cortex and the striatum in the computation of goal values and prediction errors. J Neurosci. 2008;28(22):5623-30. Epub 2008/05/30. doi: 28/22/5623 [pii]10.1523/JNEUROSCI.1309-08.2008. PubMed PMID: 18509023.

20.	Tobler PN, Fletcher PC, Bullmore ET, Schultz W. Learning-related human brain activations reflecting individual finances. Neuron. 2007;54(1):167-75. Epub 2007/04/06. doi: S0896-6273(07)00181-X [pii]10.1016/j.neuron.2007.03.004. PubMed PMID: 17408585.

21.	Klein-Flugge MC, Hunt LT, Bach DR, Dolan RJ, Behrens TE. Dissociable reward and timing signals in human midbrain and ventral striatum. Neuron. 2011;72(4):654-64. Epub 2011/11/22. doi: S0896-6273(11)00779-3 [pii]10.1016/j.neuron.2011.08.024. PubMed PMID: 22099466; PubMed Central PMCID: PMC3219831.

22.	Hein G, Engelmann JB, Vollberg MC, Tobler PN. How learning shapes the empathic brain. Proc Natl Acad Sci U S A. 2016;113(1):80-5. doi: 10.1073/pnas.1514539112. PubMed PMID: 26699464; PubMed Central PMCID: PMCPMC4711838.

23.	Rutledge RB, Dean M, Caplin A, Glimcher PW. Testing the reward prediction error hypothesis with an axiomatic model. J Neurosci. 2010;30(40):13525-36. doi: 10.1523/JNEUROSCI.1747-10.2010. PubMed PMID: 20926678; PubMed Central PMCID: PMCPMC2957369.

24.	Tinklepaugh OL. An experimental study of representative factors in monkeys. Journal of Comparative Psychology. 1928;8:197-236.

25.	Burke KA, Franz TM, Miller DN, Schoenbaum G. The role of the orbitofrontal cortex in the pursuit of happiness and more specific rewards. Nature. 2008;454(7202):340-4. Epub 2008/06/20. doi: nature06993 [pii]10.1038/nature06993. PubMed PMID: 18563088.

26.	Murray EA, Rudebeck PH. Specializations for reward-guided decision-making in the primate ventral prefrontal cortex. Nat Rev Neurosci. 2018;19(7):404-17. doi: 10.1038/s41583-018-0013-4. PubMed PMID: 29795133.

27.	Rudebeck PH, Saunders RC, Lundgren DA, Murray EA. Specialized Representations of Value in the Orbital and Ventrolateral Prefrontal Cortex: Desirability versus Availability of Outcomes. Neuron. 2017;95(5):1208-20 e5. doi: 10.1016/j.neuron.2017.07.042. PubMed PMID: 28858621; PubMed Central PMCID: PMCPMC5600902.

28.	Murray EA, Moylan EJ, Saleem KS, Basile BM, Turchi J. Specialized areas for value updating and goal selection in the primate orbitofrontal cortex. eLife. 2015;4. doi: 10.7554/eLife.11695. PubMed PMID: 26673891; PubMed Central PMCID: PMCPMC4739757.

29.	Lopatina N, McDannald MA, Styer CV, Peterson JF, Sadacca BF, Cheer JF, et al. Medial Orbitofrontal Neurons Preferentially Signal Cues Predicting Changes in Reward during Unblocking. J Neurosci. 2016;36(32):8416-24. doi: 10.1523/JNEUROSCI.1101-16.2016. PubMed PMID: 27511013; PubMed Central PMCID: PMCPMC4978801.

30.	Lopatina N, McDannald MA, Styer CV, Sadacca BF, Cheer JF, Schoenbaum G. Lateral orbitofrontal neurons acquire responses to upshifted, downshifted, or blocked cues during unblocking. eLife. 2015;4. doi: 10.7554/eLife.11299. PubMed PMID: 26670544; PubMed Central PMCID: PMCPMC4733037.

31.	Watanabe M. Reward expectancy in primate prefrontal neurons. Nature. 1996;382:629-32.

32.	Dickinson A. Actions and habits: the development of behavioural autonomy. Philosophical Transactions of the Royal Society of London B, Biological Sciences. 1985;108:67-78.

33.	Niv Y, Schoenbaum G. Dialogues on prediction errors. Trends Cogn Sci. 2008;12(7):265-72. Epub 2008/06/24. doi: S1364-6613(08)00137-X [pii]10.1016/j.tics.2008.03.006. PubMed PMID: 18567531.

34.	Chumbley JR, Burke CJ, Stephan KE, Friston KJ, Tobler PN, Fehr E. Surprise beyond prediction error. Hum Brain Mapp. 2014;35(9):4805-14. doi: 10.1002/hbm.22513. PubMed PMID: 24700400; PubMed Central PMCID: PMCPMC4312927.

35.	Fouragnan E, Retzler C, Philiastides MG. Separate neural representations of prediction error valence and surprise: Evidence from an fMRI meta-analysis. Hum Brain Mapp. 2018;39(7):2887-906. Epub 2018/03/27. doi: 10.1002/hbm.24047. PubMed PMID: 29575249.

36.	Amiez C, Joseph JP, Procyk E. Reward Encoding in the Monkey Anterior Cingulate Cortex. Cereb Cortex. 2006;16(7):1040-55. PubMed PMID: 16207931.

37.	Pearce JM, Hall G. A model for Pavlovian learning: variations in the effectiveness of conditioned but not of unconditioned stimuli. Psychol Rev. 1980;87(6):532-52. PubMed PMID: 7443916.

38.	Behrens TE, Woolrich MW, Walton ME, Rushworth MF. Learning the value of information in an uncertain world. Nat Neurosci. 2007;10(9):1214-21. Epub 2007/08/07. doi: nn1954 [pii]10.1038/nn1954. PubMed PMID: 17676057.

39.	Nassar MR, Wilson RC, Heasly B, Gold JI. An approximately Bayesian delta-rule model explains the dynamics of belief updating in a changing environment. J Neurosci. 2010;30(37):12366-78. doi: 10.1523/JNEUROSCI.0822-10.2010. PubMed PMID: 20844132; PubMed Central PMCID: PMCPMC2945906.

40.	Rushworth MF, Mars RB, Summerfield C. General mechanisms for making decisions? Curr Opin Neurobiol. 2009;19(1):75-83. Epub 2009/04/08. doi: S0959-4388(09)00007-5 [pii]10.1016/j.conb.2009.02.005. PubMed PMID: 19349160.

41.	Bromberg-Martin ES, Matsumoto M, Hikosaka O. Dopamine in motivational control: rewarding, aversive, and alerting. Neuron. 2010;68(5):815-34. doi: 10.1016/j.neuron.2010.11.022. PubMed PMID: 21144997; PubMed Central PMCID: PMCPMC3032992.

42.	Schultz W. Muliple reward signals in the brain. Nature Reviews Neuroscience. 2000;1:199-207.

43.	Glascher JP, O'Doherty JP. Model-based approaches to neuroimaging: combining reinforcement learning theory with fMRI data. Wiley Interdiscip Rev Cogn Sci. 2010;1(4):501-10. doi: 10.1002/wcs.57. PubMed PMID: 26271497.

44.	Rushworth MF, Noonan MP, Boorman ED, Walton ME, Behrens TE. Frontal cortex and reward-guided learning and decision-making. Neuron. 2011;70(6):1054-69. Epub 2011/06/22. doi: S0896-6273(11)00395-3 [pii]10.1016/j.neuron.2011.05.014. PubMed PMID: 21689594.

45.	Rangel A, Camerer C, Montague PR. A framework for studying the neurobiology of value-based decision making. Nat Rev Neurosci. 2008;9(7):545-56. Epub 2008/06/12. doi: nrn2357 [pii]10.1038/nrn2357. PubMed PMID: 18545266.

46.	Daw ND, Gershman SJ, Seymour B, Dayan P, Dolan RJ. Model-based influences on humans' choices and striatal prediction errors. Neuron. 2011;69(6):1204-15. Epub 2011/03/26. doi: S0896-6273(11)00125-5 [pii]10.1016/j.neuron.2011.02.027. PubMed PMID: 21435563; PubMed Central PMCID: PMC3077926.

47.	Wilson RC, Niv Y. Is Model Fitting Necessary for Model-Based fMRI? PLoS Comput Biol. 2015;11(6):e1004237. Epub 2015/06/19. doi: 10.1371/journal.pcbi.1004237. PubMed PMID: 26086934; PubMed Central PMCID: PMCPMC4472514.
